# Supplementary figures and images for: The impact of 8-week re-training following a 14-week period of training cessation on Greco-Roman Wrestlers
Source: PLoS One. 2025 Jun 25;20(6):e0326731. doi: 10.1371/journal.pone.0326731 (PMC12194023; doi:10.1371/journal.pone.0326731)

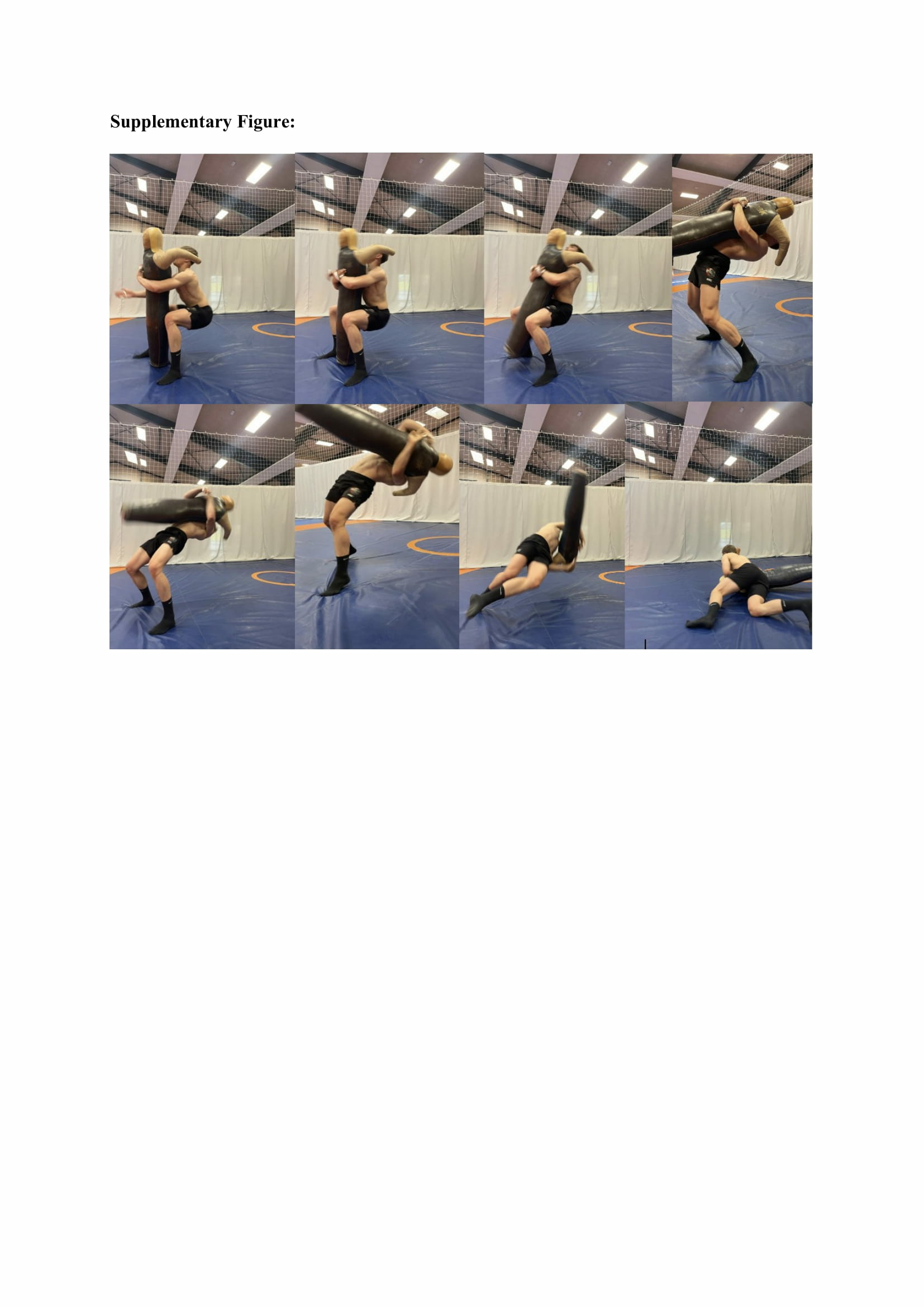

Supplement: S1 — (TIF) [file pone.0326731.s001.tif]
