# Supplementary material for: The impact of 8-week re-training following a 14-week period of training cessation on Greco-Roman Wrestlers
Source: PLoS One. 2025 Jun 25;20(6):e0326731. doi: 10.1371/journal.pone.0326731 (PMC12194023; doi:10.1371/journal.pone.0326731)
Supplement: Attachment — Submitted filename: PONE-D-25-10641_R2.pdf [file pone.0326731.s004.pdf]

PLOS ONE

The impact of 8-week re-training following a 14-week period of training cessation on Greco-Roman Wrestlers

--Manuscript Draft--

**Manuscript Number:**	PONE-D-25-10641R2
**Article Type:**	Research Article
**Full Title:**	The impact of 8-week re-training following a 14-week period of training cessation on Greco-Roman Wrestlers
**Short Title:**	RETURN TO COMPETITION FROM PANDEMIC
**Corresponding Author:**	Tuba Melekoglu, Ph.D.<br>Akdeniz University Faculty of Sport Sciences<br>Antalya, Antalya TÜRKIYE
**Keywords:**	Wrestling; Training; training cessation; Performance; Heart Rate Variability
**Abstract:**	Background This study aimed to examine the changes in physical and physiological conditions in elite wrestlers from the Turkish National Wrestling Team, who experienced 14 weeks of restricted physical activity during the COVID-19 lockdown, followed by an 8-week period of retraining and competition.<br>Methods Twenty male elite wrestlers from the National Greco-Roman Wrestling Team participated in the research. Heart Rate Variability values were measured during the training cessation period and for 8 weeks of subsequent training and then interpreted for training periods with different workloads. Body fat percentage values, initially measured during training cessation, were recorded at 2-week intervals during the training period. To determine the fitness status of wrestlers, the Specific Wrestling Fitness Test was used before and following the 8 weeks of training period.<br>Results A gradual decrease in both body fat percentage and weight was observed throughout the course of the training period. The SWFT scores showed significant improvements (31.40 ±2.91 vs. 37.40 ±3.22) following the training period. Heart rate variability decreased during the detraining period, progressively improved throughout the 8-week retraining, and subsequently declined during the competition phase, reaching levels similar to those observed during training cessation.<br>Conclusions Our results suggest that athletes undergo identical reactions in their autonomous nervous system during both competition and training cessation period. Obtaining a comprehensive understanding of these changes can enable coaches and athletes to make accurate decisions in order to optimize training adaptations and attain overall athletic success. Furthermore, over a period of eight weeks following a long non-training period, significant improvements in athletes' body fat, muscle mass and wrestling performance can be achieved along with training. Moreover, HRV monitoring revealed that autonomic nervous system balance was compromised during both the 14-week detraining and the subsequent competition phases, underlining the need for careful training load management to optimize recovery and performance readiness.
**Order of Authors:**	Burhan DEMİRKIRAN
	Ali IŞIN
	Yılmaz SUNGUR
	Tuba Melekoglu, Ph.D.
**Opposed Reviewers:**	
**Response to Reviewers:**	Reviewer #2:<br>We sincerely thank you for your valuable comments and suggestions. We have carefully addressed each of your points and revised the manuscript accordingly. Below, we provide a detailed, point-by-point response. The authors' responses are written in italics. Where appropriate, changes made in the manuscript have been highlighted in yellow.<br>Suggestion: Page 18, line 26 – authors failed to report how long the detraining phase was in the abstract section. If you state that retraining period was 8 weeks, it would be of great value to also know how long was the detraining phase.

Powered by Editorial Manager® and ProduXion Manager® from Aries Systems Corporation

	Response: Thank you for your valuable comment. As you rightly pointed out, clearly stating the duration of the detraining phase adds clarity and value to the abstract and overall interpretation. Although the 14-week detraining period was already mentioned in the background section in abstract, we have now revised the conclusion section to explicitly include the detraining duration in the relevant sentence for improved clarity. The revised sentence now reads:<br>“Moreover, HRV monitoring revealed that autonomic nervous system balance was compromised during both the 14-week detraining and the subsequent competition phases, underlining the need for careful training load management to optimize recovery and performance readiness.”<br>We hope this revision addresses your concern.<br>Suggestion: Page 19, line 44 – “During these periods of partial or complete training interruption, it has been suggested that the organism’s responsiveness to exercise is significantly prolonged”. I am not sure what this sentence means. Refine for clarity.<br>Response: Thank you for your comment. We agree that the original sentence lacked clarity and could be misinterpreted. To improve the precision of the statement, we have revised the sentence on Page 19, line 44 as follows:<br>“During periods of partial or complete training interruption, the body’s physiological responsiveness to exercise stimuli may become diminished or delayed, leading to a slower return to previous performance levels upon retraining.”<br>We believe this updated version more accurately reflects the intended meaning and appreciate your helpful suggestion.<br><br>Suggestion: Page 19, line 46 – “Athletes facing training interruptions may encounter risk factors that negatively impact performance, including declines in muscle strength, muscle size, cardiovascular endurance, as well as impairments in neuromuscular coordination and flexibility.” Provide citation(s).<br>Response: We have added relevant citations to the sentence on Page 19, line 46, which now reads:<br><br>“Athletes facing training interruptions may encounter risk factors that negatively impact performance, including declines in muscle strength, muscle size, cardiovascular endurance, as well as impairments in neuromuscular coordination and flexibility (Alvurdu et al., 2022; Bosquet et al., 2013; Mujika & Padilla, 2000b).<br><br>Suggestion: Page 21, line 79 – “Proper training methods can minimize the risk of injury, prevent long-term losses in muscle size and strength, and facilitate a faster and more reliable return to optimal fitness and performance levels. Structured and individualized retraining programs are crucial for minimizing injury risk and accelerating performance recovery in elite 75 wrestlers following prolonged periods of training cessation. Given the detrimental effects of training cessation on athletic performance, it is essential for athletes to implement appropriate strategies to mitigate performance declines.” Move this section to discussion. It deviates from the key points in the Introduction and it makes too long.<br>Response: As recommended, we have moved the relevant section from the Introduction to the Discussion to maintain a clearer focus on the key points and improve the overall structure and flow of the manuscript. We believe this adjustment enhances both the coherence and readability of the paper.<br><br>Suggestion: Page 24, line 148 – which device did you use to measure HRV?<br>Response: We have revised the sentence to include the specific device used for HRV measurement. The updated sentence now reads:<br>“HRV data were collected using a Polar H10 heart rate chest strap sensor.”<br><br>Suggestion: Page 24, line 157 – Turkish national team I’d assume? Mention this. It’s important.<br>Response: We have revised the sentence to clearly indicate that the participants were members of the Turkish National Wrestling Team. The updated sentence now reads:<br>“This study included 20 male volunteer wrestlers (aged 21.75 ± 3.11 years) who were members of the Turkish National Wrestling Team and had been training for at least 10 years.”<br><br>Suggestion: Page 26, line 199 – they COULD not… Stay in the past tense.<br>Response: As suggested, we have revised the sentence to remain consistent with the

	past tense. The corrected version now reads:<br>“Due to the constraints imposed by the pandemic, the wrestlers unable to perform a structured training program during the long-term training cessation period.”<br><br>Suggestion: Page 26, line 213 – WERE required. The same principle applies.<br>Response: As suggested, we have revised the sentence to remain consistent with the past tense. The corrected version now reads:<br>“Wrestlers were required to attend at least 90 % of the training sessions. The weekly breakdown of the 8-week training program is provided in Table 1.”<br><br>Suggestion: Page 28, line 239 – “The participant was performed three sets”.<br>Response: As suggested, we have revised the sentence to remain consistent with the past tense. The corrected version now reads:<br>“Each participant performed three sets of 30-second dummy throws, separated by 20-second rest intervals.”<br><br>Suggestion: As I said in the initial review, this manuscript would benefit greatly if the native English-speaking person reads it.<br>Response: As per your suggestion, the manuscript has been thoroughly reviewed and revised by a native English speaker to improve its linguistic quality, clarity, and fluency.”
**Additional Information:**	
**Question**	**Response**
**Financial Disclosure**<br><br>Enter a financial disclosure statement that describes the sources of funding for the work included in this submission. Review the submission guidelines for detailed requirements. View published research articles from *PLOS ONE* for specific examples.<br><br>This statement is required for submission and **will appear in the published article** if the submission is accepted. Please make sure it is accurate.<br><br>**Funded studies**<br>Enter a statement with the following details:<br>• Initials of the authors who received each award<br>• Grant numbers awarded to each author<br>• The full name of each funder<br>• URL of each funder website<br>• Did the sponsors or funders play any role in the study design, data collection and analysis, decision to publish, or preparation of the manuscript?<br><br>Did you receive funding for this work?	Yes

Please add funding details.<br>as follow-up to "**Financial Disclosure**<br><br>Enter a financial disclosure statement that describes the sources of funding for the work included in this submission. Review the submission guidelines for detailed requirements. View published research articles from *PLOS ONE* for specific examples.<br><br>This statement is required for submission and **will appear in the published article** if the submission is accepted. Please make sure it is accurate.<br><br>**Funded studies**<br>Enter a statement with the following details:<br>• Initials of the authors who received each award<br>• Grant numbers awarded to each author<br>• The full name of each funder<br>• URL of each funder website<br>• Did the sponsors or funders play any role in the study design, data collection and analysis, decision to publish, or preparation of the manuscript?<br><br>Did you receive funding for this work?"	Initials of author: TM<br>Project Number: TYL-2021-5677<br>Funder:Scientific Research Projects Coordination Unit of Akdeniz University.<br>Website: https://bap.akdeniz.edu.tr/tr<br>Sponsors or funders didn't play any role in the study design, data collection and analysis, decision to publish, or preparation of the manuscript.
Please select the country of your main research funder (please select carefully as in some cases this is used in fee calculation).<br>as follow-up to "**Financial Disclosure**<br><br>Enter a financial disclosure statement that describes the sources of funding for the work included in this submission. Review the submission guidelines for detailed requirements. View published research articles from *PLOS ONE* for specific examples.<br><br>This statement is required for submission and **will appear in the published article** if the submission is accepted. Please make sure it is accurate.	TURKEY - TR

**Funded studies**<br>Enter a statement with the following details:<br>• Initials of the authors who received each award<br>• Grant numbers awarded to each author<br>• The full name of each funder<br>• URL of each funder website<br>• Did the sponsors or funders play any role in the study design, data collection and analysis, decision to publish, or preparation of the manuscript?<br><br>Did you receive funding for this work?"	
**Competing Interests**<br><br>Use the instructions below to enter a competing interest statement for this submission. On behalf of all authors, disclose any competing interests that could be perceived to bias this work—acknowledging all financial support and any other relevant financial or non-financial competing interests.<br><br>This statement is required for submission and **will appear in the published article** if the submission is accepted. Please make sure it is accurate and that any funding sources listed in your Funding Information later in the submission form are also declared in your Financial Disclosure statement.<br><br>View published research articles from *PLOS ONE* for specific examples.	The authors have declared that no competing interests exist.

**NO authors have competing interests**<br><br>Enter: *The authors have declared that no competing interests exist.*<br><br>**Authors with competing interests**<br><br>Enter competing interest details beginning with this statement:<br><br>*I have read the journal's policy and the authors of this manuscript have the following competing interests: [insert competing interests here]*<br><br>* typeset	
**Ethics Statement**<br><br>Enter an ethics statement for this submission. This statement is required if the study involved:<br><br>• Human participants<br>• Human specimens or tissue<br>• Vertebrate animals or cephalopods<br>• Vertebrate embryos or tissues<br>• Field research<br><br>Write "N/A" if the submission does not require an ethics statement.<br><br>General guidance is provided below. Consult the submission guidelines for detailed instructions. **Make sure that all information entered here is included in the Methods section of the manuscript.**	Name of the institutional ethics committee: Akdeniz University Clinical Research Ethics Committee<br><br>Approval Number: KAEK-758 / 08.10.2020<br><br>A written consent obtained from all of the participants

**Format for specific study types**<br><br>**Human Subject Research (involving human participants and/or tissue)**<br>• Give the name of the institutional review board or ethics committee that approved the study<br>• Include the approval number and/or a statement indicating approval of this research<br>• Indicate the form of consent obtained (written/oral) or the reason that consent was not obtained (e.g. the data were analyzed anonymously)<br><br>**Animal Research (involving vertebrate animals, embryos or tissues)**<br>• Provide the name of the Institutional Animal Care and Use Committee (IACUC) or other relevant ethics board that reviewed the study protocol, and indicate whether they approved this research or granted a formal waiver of ethical approval<br>• Include an approval number if one was obtained<br>• If the study involved *non-human primates*, add *additional details* about animal welfare and steps taken to ameliorate suffering<br>• If anesthesia, euthanasia, or any kind of animal sacrifice is part of the study, include briefly which substances and/or methods were applied<br><br>**Field Research**<br><br>Include the following details if this study involves the collection of plant, animal, or other materials from a natural setting:<br>• Field permit number<br>• Name of the institution or relevant body that granted permission	
**Data Availability**<br><br>Authors are required to make all data underlying the findings described fully available, without restriction, and from the time of publication. PLOS allows rare exceptions to address legal and ethical concerns. See the PLOS Data Policy and FAQ for detailed information.	Yes - all data are fully available without restriction

A Data Availability Statement describing where the data can be found is required at submission. Your answers to this question constitute the Data Availability Statement and **will be published in the article**, if accepted.<br><br>**Important:** Stating 'data available on request from the author' is not sufficient. If your data are only available upon request, select 'No' for the first question and explain your exceptional situation in the text box.<br><br>Do the authors confirm that all data underlying the findings described in their manuscript are fully available without restriction?	
**Describe where the data may be found in full sentences. If you are copying our sample text, replace any instances of XXX with the appropriate details.**<br><br>• If the data are **held or will be held in a public repository**, include URLs, accession numbers or DOIs. If this information will only be available after acceptance, indicate this by ticking the box below. For example: *All XXX files are available from the XXX database (accession number(s) XXX, XXX.*).<br>• If the data are all contained **within the manuscript and/or Supporting Information files**, enter the following: *All relevant data are within the manuscript and its Supporting Information files.*<br>• If neither of these applies but you are able to provide **details of access elsewhere**, with or without limitations, please do so. For example:<br><br>*Data cannot be shared publicly because of [XXX]. Data are available from the XXX Institutional Data Access / Ethics Committee (contact via XXX) for researchers who meet the criteria for access to confidential data.*<br><br>*The data underlying the results presented in the study are available from (include the name of the third party*	All dataset files are available from the figshare database (http://figshare.com/articles/dataset/HRV_and_Antropometrical_Dataset/29047097/1)

and contact information or URL).<br>• This text is appropriate if the data are owned by a third party and authors do not have permission to share the data.<br><br>* typeset	
Additional data availability information:	

28-05-2025
Editor in Chief
PLOS ONE

Dear Editor,

We sincerely thank you for the opportunity to revise and resubmit our manuscript (Submission ID: PONE-D-25-10641), entitled "The Impact of 8-Week Re-Training Following a 14-Week Period of Training Cessation on Greco-Roman Wrestlers."

We greatly appreciate the constructive feedback and valuable recommendations provided by the reviewer 2. In response, we have carefully addressed each comment in detail and made substantial revisions to enhance the clarity, scientific rigor, and overall presentation of the manuscript. Our detailed responses to each point raised by the reviewer are provided below, and the corresponding changes have been clearly highlighted in the revised manuscript.

All authors have reviewed and approved the revisions, and I have been designated as the corresponding author for further communication.

We believe that the improvements made in response to the reviewers' and editor's suggestions have significantly strengthened the manuscript. We hope that the revised version meets the publication standards of PLOS ONE.

Thank you very much for your time and consideration.

Yours sincerely,

Tuba Melekoğlu
Corresponding Author on behalf of all co-authors

Akdeniz University, Faculty of Sport Sciences
tmelekoglu@akdeniz.edu.tr

Manuscript
Click here to access/download;Manuscript;Manuscript- Rev 2-without marking.docx

The impact of 8-week re-training following a 14-week period of training cessation on Greco-Roman Wrestlers

Burhan DEMİRKIRAN[1,2], Ali IŞIN[1,3], Yılmaz SUNGUR[1,4], Tuba MELEKOĞU [1,5*]

[1] Department of Coaching Education, Faculty of Sport Sciences, Akdeniz University, Antalya, Turkey
[2] 0000-0001-7447-627X (ORCID)
[3] 0000-0003-4666-2117 (ORCID)
[4] 0000-0002-0833-051X (ORCID)
[5] 0000-0001-9223-8849 (ORCID)

*Corresponding author: Tuba Melekoğlu, Akdeniz University, Faculty of Sports Sciences, 07070, Antalya/Turkey. phone: +90 242 53196828 e-mail: tmelekoglu@akdeniz.edu.tr

Declarations

Content for publication

Not applicable

Competing interests

The authors declare that they have no competing interests.

Funding

This work was supported by The Scientific Research Projects Coordination Unit of Akdeniz University. Project Number: TYL-2021-5677

Authors' contributions

TM and BD: conceptualization, methodology & data curation; TM: funding acquisition; BD, AI, YS: investigation; TM: supervision; TM, BD, AI, YS: writing - original draft; AI, TM: review & editing.

Acknowledgements

The authors would like to thank the participants for volunteering their time and the Türkiye National Greco-Roman Wrestling Team.

Data availability statement

The data are publicly available at:

https://akdeniz-my.sharepoint.com/:x:/g/personal/tmelekoglu_akdeniz_edu_tr/ERw7tnPaEFlPijwn5fNHUm0B3FSGgQio-tw2uubqm19BgA?e=lYhzkK

https://akdeniz-my.sharepoint.com/:x:/g/personal/tmelekoglu_akdeniz_edu_tr/EZKbAa0dMTlMlyZUE54SUDEBFoduS7qR5dytS-6ukSmnbA?e=oo8oVW

Abstract

Background This study aimed to examine the changes in physical and physiological conditions in elite wrestlers from the Turkish National Wrestling Team, who experienced 14 weeks of restricted physical activity during the COVID-19 lockdown, followed by an 8-week period of retraining and competition.

Methods Twenty male elite wrestlers from the National Greco-Roman Wrestling Team participated in the research. Heart Rate Variability values were measured during the training cessation period and for 8 weeks of subsequent training and then interpreted for training periods with different workloads. Body fat percentage values, initially measured during training cessation, were recorded at 2-week intervals during the training period. To determine the fitness status of wrestlers, the Specific Wrestling Fitness Test was used before and following the 8 weeks of training period.

Results A gradual decrease in both body fat percentage and weight was observed throughout the course of the training period. The SWFT scores showed significant improvements (31.40 ±2.91 vs. 37.40 ±3.22) following the training period. Heart rate variability decreased during the detraining period, progressively improved throughout the 8-week retraining, and subsequently declined during the competition phase, reaching levels similar to those observed during training cessation.

Conclusions Our results suggest that athletes undergo identical reactions in their autonomous nervous system during both competition and training cessation period. Obtaining a comprehensive understanding of these changes can enable coaches and athletes to make accurate decisions in order to optimize training adaptations and attain overall athletic success. Furthermore, over a period of eight weeks following a long non-training period, significant improvements in athletes' body fat,

muscle mass and wrestling performance can be achieved along with training. Moreover, HRV monitoring revealed that autonomic nervous system balance was compromised during both the 14-week detraining and the subsequent competition phases, underlining the need for careful training load management to optimize recovery and performance readiness.

Keywords: Wrestling, training, training cessation, performance, heart rate variability.

Introduction

Elite athletes engage in continuous, high-intensity training that is planned and systematic. This approach is essential for achieving success in international competitions, sustaining achievements, and maintaining peak performance over extended periods. As a result of these high-intensity training regimens, the physiological adaptations observed in elite athletes differ significantly from those seen in sedentary individuals and less-trained athletes. Therefore, athletes who aim to perform at the highest level not only seek to further develop these adaptations but also strive to maintain them. To preserve or enhance these physiological adaptations, it is essential to apply training stimuli at a consistent and adequate level. While excessive training stimuli may lead to overtraining, insufficient or completely discontinued training can result in performance declines known as detraining. [1, 2].

High-level competitive athletes who wish to avoid performance loss typically minimize the duration of detraining by limiting breaks from training. However, in certain circumstances—such as injury, chronic conditions, or pregnancy in female athletes— athletes may be forced to stop training. During periods of partial or complete training interruption, the body’s physiological responsiveness to exercise stimuli may become diminished or delayed, leading to a slower return to previous performance levels upon retraining [3]. Athletes facing training interruptions may encounter risk factors that negatively impact performance, including declines in muscle strength and size, cardiovascular endurance, along with impairments in neuromuscular coordination and flexibility [3-5]. Additionally, prolonged periods of inactivity are associated with an elevated risk of musculoskeletal injuries upon return to training [6, 7]. When the interruption period lasts for approximately four weeks, reductions are primarily observed in general endurance and muscular

endurance performance, while muscle strength remains largely unaffected [8, 9]. However, if training cessation exceeds four weeks, both muscle strength and endurance show significant declines [3]. Furthermore, reductions in muscle strength and size are influenced by the duration of the detraining period. Short-term detraining typically leads to minimal or no measurable change, whereas extended detraining results in a more rapid loss of muscle size compared to maximal strength [10-13]. Research indicates that after a period of training cessation, skinfold thickness, body fat mass, and percentage tend to increase, while fat-free mass and athletic performance decrease [5].

In elite wrestling, multiple performance components are required to execute consecutive high-intensity physical efforts. Flexibility, agility, reaction time, and the execution of wrestling-specific techniques form the foundation of these components. These attributes are essential for the effective application of strength during performance [14]. Following a 10-week training break in wrestlers, concentric and eccentric isokinetic strengths of the knee and shoulder flexor and extensor were assessed at various angular velocities. Significant decreases in isokinetic muscle strength were detected at angular velocities of 60°, 180°, and 300° [6]. Such reductions not only impair performance but also increase injury risk. In another study, a significant reduction in flexibility and agility was observed among collegiate wrestlers aged 18–21 years after a 10-week training cessation, regardless of changes in muscle mass percentage [14].

Several studies have shown that athletes can regain lost performance relatively quickly following short-term interruptions [15, 16]. Moreover, Valenzuela et al. [17] emphasized that inappropriate or abrupt return-to-play protocols may lead to overuse injuries and compromise full recovery, underscoring the importance of gradual and individualized training resumption. The literature also suggests that muscle strength can be restored rapidly after short-term (~three-week) periods of inactivity. Yasuda, Loenneke (16) reported that individuals with prior strength training experience

were able to regain muscular strength through blood flow restriction training after a three-week detraining phase.

In addition to physical performance losses such as reduced strength and endurance, the autonomic nervous system (ANS)—a vital marker of general health and a determinant of athletic performance—may also be adversely affected by training cessation. Heart rate variability (HRV), which is frequently used as a non-invasive marker of ANS function, can exhibit significant deterioration following prolonged periods of inactivity. Sustained training interruptions are commonly associated with decreased HRV values, which may contribute to reductions in performance capacity [18].

In early 2020, the global outbreak of coronavirus disease (COVID-19) was declared a public health emergency of international concern. In response, social gatherings were restricted and training facilities were closed, causing widespread disruptions in athletic preparation. The impact on elite athletes was substantial, with ongoing championships suspended and major international events postponed.

Moreover, most elite athletes were compelled to train alone in isolated and often unsupervised environments, such as their homes. Upon the easing of lockdown restrictions, it was recommended that training programs be carefully structured and that athletes' performance and health parameters be closely monitored to ensure a safe return to competition. This approach helped minimize injury risk and supported a gradual recovery of physical condition [19, 20].

Although several studies have examined the effects of prolonged inactivity on physical and physiological adaptations and performance in elite athletes, there is a lack of research specifically investigating these effects in elite wrestlers. In particular, little is known about the physiological recovery trajectory following 14 weeks of inactivity in this population. Furthermore, the duration

required for elite wrestlers to fully regain optimal performance capacity in preparation for competition remains insufficiently understood [15, 21, 22].

The purpose of this study was to examine the changes in the physical and physiological parameters in elite wrestlers on the Turkish National Wrestling Team who underwent a 14-week period of limited and unstructured activity during the COVID-19 lockdown, followed by an 8-week retraining and competition phase.

Materials and Methods

Experimental Approach to the Problem

This study is a longitudinal investigation that aims to examine the potential alterations in the physical, physiological, and performance metrics of elite senior national wrestlers competing in the Greco-Roman style. These wrestlers had not engaged in field-based training for a minimum of 14 weeks due to the Covid-19 epidemic. The study focuses on their performance outcomes and autonomous system responses following the resumption of field training. The recruitment period for the study was between 15.10.2020 and 15.02.2021.

During the 14-week COVID-19 lockdown period, information regarding the wrestlers' physical activity levels was obtained through self-reports collected by the Turkish National Wrestling Team coaches. Notably, one of the coaches involved in data collection is also listed as an author of this study, which provided close oversight and contributed to the reliability of the self-reported data. The athletes reported that they remained at home in accordance with pandemic restrictions and engaged only in basic home-based exercises, walking, and limited jogging. They did not participate in any structured or externally supervised training programs, and no specific dietary interventions were implemented. In the early phase of the lockdown, the wrestlers were predominantly inactive due to the uncertainty surrounding the duration of the restrictions. As the lockdown persisted, they

gradually began to engage in limited individual exercises. Nevertheless, these activities lacked the structured intensity, volume, frequency, and duration characteristic of systematic athletic training. HRV is a well-established non-invasive indicator of ANS regulation, specifically reflecting the balance between sympathetic and parasympathetic nervous system activities. Higher HRV is generally associated with increased parasympathetic (vagal) modulation and improved cardiovascular adaptability, whereas reduced HRV reflects sympathetic dominance and impaired autonomic flexibility. [23] In this study, HRV was measured to assess the wrestlers' general health and fitness status during the 8-week retraining period following the training cessation period, and also during the competition period that followed. HRV data were collected using a Polar H10 heart rate chest strap sensor.

To evaluate ANS through HRV, the following commonly used parameters were recorded: standard deviation of NN intervals (SDNN), root mean square of successive RR interval differences (RMSSD), peak frequency of the high-frequency band (HF), peak frequency of the low-frequency band (LF), and the LF/HF power ratio. HRV measurements, averaged over at least 10 days during the training cessation period, were obtained through 5-minute recordings taken while lying in bed immediately upon waking, each morning for 8 weeks after the retraining began.

The athletes' body weight, subcutaneous fat thickness, and body fat percentage were assessed on the first day they of training and subsequently at 2-week intervals throughout the retraining period. In addition, the athletes completed the Specific Wrestling Fitness Test (SWFT), a wrestling-specific assessment, on the first day of retraining and again after eight weeks. Blood lactate samples were collected immediately before, immediately after, and four minutes post-test to determine whether the SWFT was performed at the intended physiological intensity.

Participants

This study included 20 male volunteer wrestlers (aged 21.75 ± 3.11 years) who were members of the Turkish National Wrestling Team and had been training for at least 10 years. The wrestlers included in the study were elite-level athletes who were actively competing prior to the training cessation period. While organized competitions and group training sessions were cancelled during the COVID-19 pandemic, these athletes maintained limited, individualized training routines adapted to the imposed restrictions. However, these activities lacked the structured intensity, volume, frequency, and duration typically required for systematic athletic development. Two of the participants had previously won medals at the European and World Championships, and an additional seven athletes had achieved national and international medals in the year preceding the training cessation. Following the cessasion period, one wrestler earned a silver medal at the World Championships and a gold medal the following year. Additionally, six athletes secured medals at European and World Championship events in the seasons following the cessation. The exceptional achievements of the participants, both prior to and following the training cessation period, clearly demonstrate their status as highly competitive elite wrestlers on the international stage.

The inclusion criteria for this study were as follows: being 19 years of age or older; being a national-level athlete with international competition experience; having similar lifestyle habits and sporting backgrounds; not taking any medication that could affect cardiac function; and having no known medical conditions that could impair cardiac or ANS function.

The exclusion criteria included: voluntary withdrawal from the study, failure to regularly attend scheduled measurement sessions, or sustaining an injury or a long-term illness during the training period.

All participants underwent a similar experience during the mandatory stay-at-home period, began retraining at the same time, and followed an identical training program throughout the retraining period. The study design and timeline are illustrated in Figure 1.

All participants provided written informed consent prior to participation. The study was conducted in accordance with the principles of the Declaration of Helsinki. This research was approved by the Akdeniz University Clinical Research Ethics Committee (KAEK-758 / 08.10.2020), and formal permission was obtained from the Wrestling Federation of Türkiye for the measurements.

Figure 1. Trial Design (Approximately Here)

Anthropometric assessments

To evaluate the changes in the physiological characteristics of the participants, body weight (BW), body fat percentage (BFP), body muscle mass (BMM) and body mass index (BMI), measurements were assessed on the last day of training cessation period and 2-week intervals during the 8-week retraining period. Measurements were conducted using a bioelectrical impedance analysis device (Tanita Body Composition Analyzer Type SC-330).

BW, subcutaneous fat thickness (SFT), BFP measurements were measured immediately after training cessation, on the first day of the retraining, and at 2-week intervals thereafter. BMI was calculated using the formula of BMI = Weight (kg)/Height^2 (m^2). BMI values were reported for descriptive purposes only and were complemented with direct body composition measurements such as subcutaneous fat thickness (SFT) to better reflect the athletes' physical profiles. Height, weight, BMI, BFP values were recorded using the integrated printer on the body composition analyzer.

Training program: Due to the constraints imposed by the pandemic, the wrestlers unable to perform a structured training program during the long-term training cessation period. In accordance with the limitations of the COVID-19 conditions, training during this time consisted of basic strength exercises, individual running and walking routines and household-item assisted workouts

that athletes could conduct at home. This period included only self-directed exercise, and no training program supervised by external trainers was implemented.

The retraining period consisted of standardized wrestling training sessions designed and monitored by the national team coaches, conducted at varying intensities over 8 weeks. Following the training cessation period (TCp), training intensity was progressively increased during the first 5 weeks (training period (Tp)). In the 6th and 7th week, training reached its peak (high intensity training period (HTp)). In the 8th week, leading into competition, training intensity was reduced to allow for tapering and recovery (form training period (FTp). After the retraining period, wrestlers participated in an international competition (competition period (Cp)).

Daily training logs were maintained to monitor compliance, and these records were regularly reviewed by the researcher (B.D.). Wrestlers were required to attend at least 90 % of the training sessions. The weekly breakdown of the 8-week training program is provided in Table 1.

Table 1. The weekly content of the 8-week training program

Week 1	Week 2	Week 3	Week 4
**Total exercise session:** 8<br>**Exercise types:**<br>**Type 1-)** 3 BST sessions (%60-70 of 1RM)<br><br>**Type 2-)** 3 WFT (1,5 hours average of each)<br><br>**Type 3-)** 2 AE sessions (1 hour running, %50-60 of HRmax, sauna)	**Total exercise session:** 10<br>**Exercise types:**<br>**Type 1-)** 4 BST sessions (%60-75 of 1RM)<br><br>**Type 2-)** 4 WFT sessions (1,5 hours average of each)<br><br>**Type 3-)** 2 AE (1 hour running, %60-70 of HRmax, sauna)	**Total exercise session:** 11<br>**Exercise types:**<br>**Type 1-)** 4 BST sessions (%70- 75 of 1RM)<br><br>**Type 2-)** 5 WFT sessions (2 hours average of each)<br><br>**Type 3-)** 1 AE session (45 minutes running, %70-75 of HRmax, sauna)	**Total exercise session:** 11<br>**Exercise types:**<br>**Type 1-)** 3 BST sessions (%70- 75 of 1RM)<br><br>**Type 2-)** 6 WFT sessions (2 hours average of each)<br><br>**Type 3-)** 1 HIIT running session (30 minutes, %70-80 of HRmax)

		**Type 4-)** 1 session core exercises	**Type 4-)** 1 session core exercises

Week 5	Week 6	Week 7	Week 8
**Total exercise session:** 10<br>**Exercise types:**<br>**Type 1-)** 3 BST sessions (%75 of 1RM)<br>**Type 2-)** 5 WFT sessions (2 hours average of each)<br>**Type 3-)** 1 AE session (1 hour running, %60-70 of HRmax, sauna)<br>**Type 4-)** 1 session core exercises	**Total exercise session:** 10<br>**Exercise types:**<br>**Type 1-)** 3 SMT session (%50-60 of 1RM)<br>**Type 2-)** 5 WFT sessions (1,5 hours average of each)<br>**Type 3-)** 1 HIIT running session (30 minutes, %70-75 of HRmax, sauna)<br>**Type 4-)** 1 HIIT running session (20 minutes, 80-90 of HRmax)	**Total exercise session:** 8<br>**Exercise types:**<br>**Type 1-)** 2 EST session (%80-90 of 1RM)<br>**Type 2-)** 5 WFT sessions (1 hour average of each)<br>**Type 3-)** 1 HIIT running session (20 minutes, 80-90 of HRmax, restitution, sauna)	**Total exercise session:** 6<br>**Exercise types:**<br>**Type 1-)** 2 EST session (%90-100 of 1RM)<br>**Type 2-)** 3 WFT sessions (30-45 minutes average of each)<br>**Type 3-)** 1 restitution trainings (30 minutes, sauna)

BST: Basic strength training, WFT: Wrestling field training, AE: Aerobic exercise, SMT: Strength maintenance training, HIIT: High intensity interval training, EST: Explosive strength training, HRmax: Maximum heart rate, 1RM: 1 Repetition maximum,

HRV measurements:

HRV was measured daily throughout the training cessation phase, which lasted 10 days prior to the start of the retraining sessions. This phase followed an 8-week training program and a 4-day competition period. Participants were provided a Polar H10 heart rate chest strap sensor (Polar Electro Oy, Kempele, Finland) with a sampling rate of 1000 Hz. A mobile application, Elite HRV, was used to record, store, and export the data.

Prior to data collection, participants were instructed on how to use their assigned HR monitor and smartphone application (Elite HRV). They were directed to measure their resting heart rate for 5 minutes each morning upon waking, while remaining in a supine position. The measurements involved evaluating the R-R intervals, with statistical analyses including SDNN and RMSSD, as well as frequency-domain analyses such as LF, HF, and LF/HF ratio [24]. Mean HRV values were calculated separately for each training phase (TCp, Tp, HTp, FTp, Cp), categorized based on training intensity.

Specific Wrestling Fitness Test (SWFT):

The SWFT was used to evaluate physical fitness capacity of the wrestlers. Participants performed the test before and after the 8-week retraining period. Blood lactate concentrations were measured before, immediately after, and 4 minutes after the SWFT using fingertip samples and a portable lactate analyzer (EDGE Blood Lactate Analyzer, Woodley Equipment Company, Lancashire).

The SWFT consists of maximum wrestling dummy throws within a timed protocol. As wrestling is a weight-classified sport, participants used dummies corresponding to their weight class:

- 22 kg dummy for athletes weighing up to 74.9 kg
- 27 kg dummy for those weighing between 75.0–89.9 kg

Each participant performed three sets of 30-second dummy throws, separated by 20-second rest intervals. After the start signal, participants attempted to throw the dummy as many times as possible within the allotted time using maximum effort [25].

The total number of throws completed across the three sets was recorded as the final test score. Prior to each test, athletes completed a 15-minute general warm-up, followed by a 5-minute specific warm-up involving wrestling dummy throws. A visual illustration of the dummy used in the SWFT is provided in Supplementary Figure 1.

Statistical analyses:

Several statistical methods were employed to test for normality, including assessments of kurtosis and skewness, histogram visualizations, Q-Q plots, and boxplots, followed by the Shapiro-Wilk test.

For comparing pre- and post-test values within groups, either the Dependent Samples t-test or the Wilcoxon signed-rank test was used, depending on data distribution.

To analyze repeated measurements over time, Repeated Measures ANOVA was conducted.

All analyses were performed using Jamovi software (version 2.3.18).

Results

Anthropometric assessments were conducted biweekly throughout the training period. Subcutaneous fat thickness was measured at the triceps, abdomen, subscapular, supraspinale, pectoral, midaxillary, and quadriceps regions using the skinfold method. Additionally, body fat percentage and body muscle mass were assessed using the bioelectrical impedance analysis (BIA) method. Statistically significant improvements were observed in both BFP and BMM parameters at each subsequent measurement point compared to the previous one. (Table 2).

Table 2. Biweekly Changes in Body Weight, Body Fat Percentage, Muscle Mass, and BMI During the Retraining Period

n = 20	W0	W2	W4	W6	W8	F	p	η2
**BW**<br>(kg)	76.49±12.27<br>70;82	75.29±11.58*<br>70;81	74.9±11.33<br>70;80	75.08±11.37<br>70;80	75.47±11.61<br>70;81	6.918	0.000	.27
**BMI**<br>(kg/m^2)	25.11±2.74<br>24;26	24.73 2.56**<br>24;26	24.60±2.48<br>23;26	24.67±2.46<br>24;26	24.78±2.44<br>24;26	7.237	0.000	.28
**BFP**<br>(%)	12.38±4.50<br>10;15	11.25±4.19***<br>9;13	10.77±3.96***<br>9;13	10.12±3.77***<br>8;12	9.66±3.66**<br>8;11	44.751	0.000	.70
**BMM**<br>(kg)	62.51±7.86<br>59;66	62.90±7.95**<br>59;67	63.25±8.11***<br>59;67	64.08±8.37***<br>60;68	65.00±8.80***<br>61;69	39.678	0.000	.68

Data in the table; presented as mean ± standard deviation (Mean ± SD) and 95% confidence interval (5%; 95% CI). *p < 0.05, **p < 0.01 ***p < 0.001 compared to the previous measurement. Statistical analysis was performed using repeated measures ANOVA followed by Bonferroni post-hoc tests. W0: pre-training period; W2: 2 weeks post-training, period; W4: 4 weeks post-training period; W6: 6 weeks post-training period; W8: 8 weeks post-training period; BW: body weight; BMI: body mass index; BFP: body fat percentage; BMM: body muscle mass; η2: partial eta squared (effect size).

There was a significant time x group interaction effect in subcutaneous fat thickness measurements. Notable reductions were observed across all assessed sites during the 8-week retraining period (Figure 2). Triceps skinfold thickness decreased significantly at each measurement point compared to the previous one ($p < 0.01$). Similarly, abdominal ($p < 0.001$), supraspinale ($p < 0.001$), midaxillary ($p < 0.01$), subscapular ($p < 0.001$), and quadriceps ($p < 0.01$) regions exhibited statistically significant reductions at every time point. In the pectoral region, a significant reduction

was observed at week 2 ($p < 0.01$) and week 4 ($p < 0.05$); however, no further significant changes were observed thereafter. These results collectively suggest a progressive improvement in body composition during the retraining phase, as evidenced by the consistent decrease in subcutaneous fat thickness across the majority of the measured anatomical sites.

Figure 2. Changes in subcutaneous fat thickness and muscle mass (Approximately here)

SWFT scores increased significantly following the 8-week training period (Table 3.). Despite the improvement in performance, post-test blood lactate levels were lower compared to pre-training values, indicating enhanced anaerobic efficiency and lactate clearance capacity. (Figure 3.).

Table 3. SWFT scores and Lactate Levels

(n = 20)	T0	T4	t	p	E
**SWFT (throws)**	31.40 ±2.91<br>30;33	37.40 ±3.22<br>36;39	10.104	0.000	2.23
			**F**	**p**	**η2**
**LApre (mmol/L)**	1.18 ± 0.33<br>1;1	1.03 ± 0.29<br>1;1	724.028	0.000	0.950
**LApost (mmol/L)**	5.21 ± 2.15<br>4;6	4.88 ± 1.55<br>4;6			
**LA4post (mmol/L)**	10.45 ± 2.84<br>9;12	8.10 ± 1.55<br>7;9			

Data in the table; presented as mean ± standard deviation (Mean ± SD) and 95% confidence interval (5%; 95% CI).
W0: pre-training period; W2: 2 weeks post-training, period; W4: 4 weeks post-training period; W6: 6 weeks post-training period; W8: 8 weeks post-training period; SWFT: specific wrestling fitness test; LApre: Lactate levels pre SWFT; LApost: Lactate levels immediately post SWFT; LA4post: Lactate levels post 4 minutes SWFT; E: effect size, η2: partial eta squared.

Table 4 and Figure 3 present the HRV variables of the participants. A significant time x group interaction was observed for HRV scores, SDNN, RMSSD, LF, LF/HF values. HRV, SDNN, LF values were significantly higher during the FTp compared to both TCp and Cp.

Table 4. HRV Values

(n = 20)	DTp	Tp	HTp	FTp	Cp	F	p	η2
**HRV**	60.80 ± 8.83<br>55;66	65.37 ±6.38<br>62;69	64.97 ± 4.63<br>62;68	70.12 ± 8.91<br>65;76	60.45 ± 6.94<br>56;65	11.384	0.002	0.487

**SDNN**	110.01 ± 55.03<br>77;143	122.76 ± 35.50<br>101;144	116.35 ± 35.02<br>95;138	131.43 ± 37.19<br>109;154	94.60 ± 25.63<br>79;110	5.330	0.001	0.308
**RMSSD**	73.49 ± 40.63<br>49;98	80.66 ± 33.85<br>60;101	75.22 ± 21.86<br>62;88	86.98 ± 27.05<br>71;103	64.06 ± 14.91<br>55;73	2.719	0.040	0.185
**LF**	2456 ± 1049<br>1821;3090	3745 ± 1638<br>2755;4735	3941±1818<br>2392;4590	3979±1775<br>2906;5052	2314±1137<br>1626;3001	6.191	0.000	0.340
**HF**	2172 ± 1161<br>1471;2874	2732 ± 1487<br>1833;3630	2757 ± 1804<br>1667;3848	2946 ± 1583<br>1989;3901	2923 ± 1691<br>907;2939	2.190	0.130	0.154
**LF/HF**	2.65 ± 0.86<br>2;3	4.04 ± 2.11<br>3;5	3.79 ± 1.71<br>3;5	4.08 ± 2.18<br>3;5	2.79 ± 1.24<br>2;4	3.162	0.022	0.209

Data in the table; presented as mean ± standard deviation (Mean ± SD) and 95% confidence interval (5%; 95% CI).
DTp :detraining period; Tp : training period ; HTp: high intensity training period; FTp: form training period ; Cp:competition period; HRV: heart rate variable score; SDNN: standard deviation of NN intervals, RMSSD: root mean square of successive RR interval differences, HF: peak frequency of the high-frequency band, LF: peak frequency of the low-frequency band (), and LF/HF: ratio of LF-to-HF power, η2: partial eta squared (effect size).

Figure 3. HRV values (Approximately here)

Discussion

This study aimed to investigate the effects of an 8-week retraining program on various physical, physiological, and athletic performance parameters, as well as on ANS tone—specifically examining the balance between the sympathetic and parasympathetic branches—in elite national team wrestlers following a 14-week period of training cessation. The findings revealed significant improvements in body composition, athletic performance, and HRV values—an established indicator of ANS function—following the 8-week retraining period.

Training cessation, or detraining, refers to the partial or complete loss of physiological adaptations previously gained through training, occurring when physical activity is reduced or stopped [3]. Short-term detraining has been reported to result in marked increases in body fat mass (+21%) and reductions in muscle mass (~4%) in elite athletes, potentially impairing performance and agility [26]. When detraining extends beyond four weeks, its negative impact on body fat percentage, muscle mass, and overall performance becomes more pronounced. If prolonged further, it may lead

to the partial or total loss of training-induced adaptations. The magnitude of these changes varies depending on the individual's initial fitness level and the duration of the detraining period. [27]. Previous studies have shown that body fat mass, body fat percentage, and skinfold thickness tend to increase, while fat-free mass and athletic performance decrease following a period of training cessation [5, 28]. In wrestling, maintaining optimal body composition—characterized by a low body fat percentage—is particularly crucial, as athletes are matched against opponents of similar body weight before each bout. A lower fat mass (FM) percentage is considered advantageous, while higher fat-free mass (FFM) is recognized as a key determinant of anaerobic performance in wrestlers. Therefore, balancing fat reduction and muscle preservation is essential for optimizing competitive readiness and athletic capability [29].

Although elite athletes may experience short-term benefits, such as super-compensation and improved recovery following a temporary reduction in training, the long-term consequences of training cessation are detrimental to their overall conditioning and performance. Elite wrestlers typically engage in three to six hours of intensive training per day to optimize their athletic capabilities. As such, their training programs must consistently target the development of strength, flexibility, and anaerobic power to maintain and enhance performance levels. [30, 31]. Under normal conditions, wrestlers take short breaks or reduce training volume and intensity for no more than three weeks after the competitive season. However, the COVID-19 pandemic forced elite athletes into an unusually prolonged period of inactivity. The pandemic led to widespread disruptions in training, limiting access to facilities and structured programs, and resulting in extended periods of detraining that negatively affected physical performance and increased the risk of injury [32, 33]. The adverse effects of prolonged training cessation include impairments in their physical condition and athletic performance, increases in body fat percentage, heightened fatigue,

a greater risk of injury, and longer recovery times [19, 20]. Even inactivity periods as short as 2–4 weeks can induce significant detraining effects, such as reductions in maximal oxygen uptake, muscle strength, neuromuscular coordination, and ventilatory efficiency. These physiological declines can increase susceptibility to injury upon return if training intensity is ramped up too quickly [3, 34].

Grazioli, Loturco (35) reported that an 8-week quarantine period negatively impacted football players' physical performance -specifically power, sprinting, body mass, and body fat mass- and emphasized the need for longer recovery timelines than those associated with regular training. Similarly, Valenzuela et al. [17] highlighted that following long-term inactivity such as during the COVID-19 lockdown, athletes require carefully structured retraining programs lasting at least 6–8 weeks to restore physiological and performance capacities.

Regardless of the cause of training interruption, previous studies have shown that the longer the inactivity period, the more pronounced the declines in endurance, strength, and neuromuscular coordination. These findings underscore the necessity of structured and individualized retraining strategies for athletes returning after extended breaks [3]. Sudden increases in training volume and intensity following confinement can overload physiological systems and elevate injury risk. Conversely, appropriate retraining approaches can minimize injury risk, prevent long-term losses in muscle size and strength, and support a more reliable return to optimal performance. Structured, individualized retraining programs are especially critical for elite wrestlers after prolonged inactivity. To mitigate performance declines and safely return to competition, athletes must implement evidence-based strategies, including progressive training loads, baseline physical assessments, and regular monitoring using tools such as HRV and perceived exertion scales. [15, 16, 36, 37].

In our study, we aimed to determine the physical and physiological adaptations that occur following an eight-week retraining program implemented after a prolonged 14-week period of inactivity. During this retraining period, a clear and progressive improvement was observed in body fat percentage, muscle mass, and athletic performance. However, as noted by Grazioli, Loturco (35) there is currently no research in the literature that allows for direct comparison regarding the time required for such improvements. Nonetheless, our findings suggest that elite wrestlers can achieve substantial gains in body composition and athletic performance within just eight weeks of training, even after an unusually extended period of inactivity.

The development of anaerobic endurance is crucial for explosive sports like wrestling. The SWFT is a widely used performance assessment tool to measure wrestlers' anaerobic endurance and overall physical fitness. The total number of wrestling dummy throws serves as an indicator of immediate physical fitness and anaerobic capacity [25]. Anaerobic endurance, maximal strength, and dynamic balance are fundamental components of wrestling performance. The rapid recovery of these parameters following a period of detraining is essential for maintaining explosive movement patterns and technical efficiency during competition. Although there is currently no study specifically examining the combined effects of training cessation and retraining periods, previous research indicates that wrestlers can improve their SWFT score after six weeks of multi-component training [30, 38]. In combat sports such as wrestling, decision-making skills and technical proficiency require practice against live opponents. Following 4–12 weeks of reduced training (~20–40% of normal load), a gradual return involving general physical preparation and progressive specific skill training over an estimated 3–5 weeks has been recommended to minimize injury risks [39].

In our study, it was observed that elite-level wrestlers significantly improved their SWFT scores by the end of the 8-week retraining period. Moreover, although the SWFT scores increased significantly, post-test lactate levels were notably lower during the retraining phase (8.10 ± 1.55 vs. 10.45 ± 2.84 mmol/L). This reduction in lactate levels, despite an increase in the number of throws performed, indicates improvements both lactate threshold and lactate clearance capacity.

Furthermore, a key finding of our study is the relationship between HRV and both training intensity and competition duration. While consistent long-term training can enhance HRV in athletes, excessively intense training may result in significant daily HRV reductions, and lower morning HRV values may reflect a decline in performance capacity. Athletes experiencing overtraining syndrome often exhibit markedly reduced HRV [40]. In addition, training cessation may also lead to a reduction in HRV. According to Ruiz-Navarro et al. [41], after five weeks of training cessation, all vagal-related HRV measures recorded before and after exercise showed a decline, indicating a deterioration in the swimmers' physical condition. The state of the ANS is influenced by accumulated physical fatigue resulting from increased training loads. Therefore, monitoring HRV can serve as an effective tool for assessing an athlete's training status and overall performance and may also be used to help prevent overtraining [40-42]. Individually tailored training load management can maximize athletic performance gains, whereas both excessive and insufficient training loads may result in detraining and accumulated fatigue. HRV monitoring has been recognized as an effective method for evaluating an athlete's physiological status in relation to sports performance [17].

In our study, HRV values were monitored throughout the eight-week retraining period and the subsequent competition phase. The data were evaluated by analyzing average HRV values across specific time intervals, categorized based on training load intensity. Overall, HRV demonstrated a

progressive improvement during the retraining phase but showed a modest decline during periods of high training intensity. Notably, during the tapering phase - when training intensity was reduced- HRV values reached their peak levels. Valenzuela, Rivas (17) suggested that the COVID-19 lockdown could have a detrimental effect on HRV in elite athletes. Similarly, Gamelin et al. [43]. reported that an eight-week period of training cessation may reverse cardiovascular autonomic adaptations previously achieved through training. On a more optimistic note, Valenzuela, Rivas (17) reported that elite athletes tend to achieve full recovery following 6 to 8 weeks of retraining, which aligns with the findings of our study. However, in our study, although HRV values progressively improved during the retraining phase, they declined again during the subsequent competition period—reaching levels comparable to those observed during the initial training cessation. This decline in HRV suggests that athletes may have experienced increased physiological stress and fatigue during competition, potentially impairing autonomic recovery. Several studies [44, 45] have demonstrated that stress and anxiety negatively influence the ANS during competitive periods, resulting in decreased HRV values. Additionally, research on recovery following injury or forced inactivity consistently shows that restoring muscle mass, cardiovascular capacity, and neuromuscular control require progressive retraining over several weeks to return to baseline levels [19]. It has also been reported that lockdown conditions caused measurable declines in aerobic fitness and neuromuscular performance among elite athletes, underscoring the universal vulnerability of this population to detraining effects, regardless of the reason for inactivity [46]. Collectively, these findings highlight the value of HRV monitoring as a non-invasive tool for assessing athletes' physiological responses to stress and anxiety during competition. Such insights can guide the development of more effective training and recovery strategies. Furthermore, implementing interventions aimed at reducing psychological stress may help maintain optimal HRV levels and support improved performance outcomes in competitive settings.

The most significant limitation of this study is the absence of a control group. After the isolation period ended, all athletes were allowed and required to resume training simultaneously. In elite athletic populations, establishing a control group is particularly challenging, as training cessation or alterations to the standard program are generally not accepted by coaches or athletes.

Another limitation is the lack of pre-pandemic or during-pandemic baseline measurements. Due to the sudden and unforeseen implementation of pandemic-related restrictions, initial assessments could not be conducted in accordance with mandatory social distancing regulations. As a result, baseline measurements were taken at the beginning of the retraining phase, and subsequent changes were tracked over time. Additionally, the study employed a convenience sampling method, as access to participants was severely restricted during the COVID-19 pandemic. Although this approach was the most practical under the given circumstances, it may limit the generalizability of the findings. Future studies are encouraged to adopt randomized or stratified sampling methods to enhance sample representativeness and strengthen the robustness of the conclusions.

Elite athletes possess a remarkable capacity to regain both athletic performance and body composition following periods of training cessation. HRV, a key marker of ANS function, fluctuates significantly in response to changes in training load and competition stress. These fluctuations underscore the importance of HRV monitoring for optimizing performance and recovery strategies.

A thorough understanding of these physiological responses can empower coaches and athletes to make more informed decisions, facilitate targeted adaptations, and ultimately achieve higher levels of athletic success. Based on the findings of our study, it is recommended that wrestlers returning from prolonged training cessation follow retraining programs that emphasize anaerobic conditioning, technical-tactical drills performed under fatigue, and gradual progression of training loads to minimize injury risk and accelerate the restoration of competitive readiness.

Conclusions

As a result of eight-week retraining period, a clear and progressive decrease in body fat percentage and an increase in muscle mass were observed. Following this training period, the Specific Wrestling Fitness Test (SWFT), which is used to assess wrestlers' physical fitness capacity, showed significant improvements in performance scores.

Throughout the retraining period, HRV values exhibited a gradual upward trend, reflecting improved autonomic balance. However, a slight decline in HRV was noted during phases of intensive training. During the tapering phase, characterized by reduced training intensity, HRV values reached their highest levels.

These findings indicate that HRV is highly sensitive to variations in training load and competitive stress. Notably, HRV values measured during the competition phase were similar to those observed during the non-training period, suggesting a potential physiological strain associated with competitive demands.

Acknowledgement

The English language, grammar, and overall clarity of this manuscript were reviewed and edited with the assistance of an advanced AI language model (ChatGPT-4o, OpenAI). Following the AI-supported editing, final revisions and adaptations were carefully made by the authors to ensure that the manuscript meets international academic writing standards and accurately reflects the intended scientific meaning.

References

1. MacInnis MJ, Gibala MJ. Physiological adaptations to interval training and the role of exercise intensity. The Journal of physiology. 2017;595(9):2915-30.

2. Schoenfeld BJ, Grgic J, Ogborn D, Krieger JW. Strength and hypertrophy adaptations between low-vs. high-load resistance training: a systematic review and meta-analysis. The Journal of Strength & Conditioning Research. 2017;31(12):3508-23.
3. Mujika I, Padilla S. Detraining: loss of training-induced physiological and performance adaptations. Part II: long term insufficient training stimulus. Sports medicine. 2000;30:145-54.
4. Bosquet L, Berryman N, Dupuy O, Mekary S, Arvisais D, Bherer L, et al. Effect of training cessation on muscular performance: A meta-analysis. Scandinavian journal of medicine & science in sports. 2013;23(3):e140-e9.
5. Alvurdu S, Baykal C, Akyildiz Z, Şenel Ö, Silva AF, Conte D, et al. Impact of prolonged absence of organized training on body composition, neuromuscular performance, and aerobic capacity: A study in youth male soccer players exposed to COVID-19 lockdown. International journal of environmental research and public health. 2022;19(3):1148.
6. Noorbakhsh M, Zarei M, Hovanloo F, Hoseini A, Yaghoubitajani Z. Exploring the influence of a 10-week specific detraining on injury risk factors among elite young wrestlers: a prospective study. Scientific Reports. 2025;15(1):7348.
7. Yamashita D, Hirata K, Yamazaki K, Mujika I, Miyamoto N. Effect of two weeks of training cessation on concentric and eccentric knee muscle strength in highly trained sprinters. Plos one. 2023;18(7):e0288344.
8. Chen Y-T, Hsieh Y-Y, Ho J-Y, Lin T-Y, Lin J-C. Two weeks of detraining reduces cardiopulmonary function and muscular fitness in endurance athletes. European Journal of Sport Science. 2022;22(3):399-406.
9. Pereira LA, Freitas TT, Pivetti B, Alcaraz PE, Jeffreys I, Loturco I. Short-term detraining does not impair strength, speed, and power performance in elite young soccer players. Sports. 2020;8(11):141.
10. Achten J, Jeukendrup AE. Heart rate monitoring: applications and limitations. Sports medicine. 2003;33:517-38.
11. Häkkinen K, Alen M, Kallinen M, Newton R, Kraemer W. Neuromuscular adaptation during prolonged strength training, detraining and re-strength-training in middle-aged and elderly people. European journal of applied physiology. 2000;83:51-62.
12. Räntilä A, Ahtiainen JP, Avela J, Restuccia J, Kidgell D, Häkkinen K. High responders to hypertrophic strength training also tend to lose more muscle mass and strength during detraining than low responders. The Journal of Strength & Conditioning Research. 2021;35(6):1500-11.
13. Halonen EJ, Gabriel I, Kelahaara MM, Ahtiainen JP, Hulmi JJ. Does Taking a Break Matter—Adaptations in Muscle Strength and Size Between Continuous and Periodic Resistance Training. Scandinavian Journal of Medicine & Science in Sports. 2024;34(10):e14739.
14. Hedya MHM. The Effect of A Detraining on some physical variables and body composition during the transition period for wrestlers. Assiut Journal of Sport Science and Arts. 2016;116(1):628-47.
15. Stokes KA, Jones B, Bennett M, Close GL, Gill N, Hull JH, et al. Returning to play after prolonged training restrictions in professional collision sports. International journal of sports medicine. 2020;41(13):895-911.
16. Yasuda T, Loenneke JP, Ogasawara R, Abe T. Effects of short-term detraining following blood flow restricted low-intensity training on muscle size and strength. Clinical physiology and functional imaging. 2015;35(1):71-5.
17. Valenzuela PL, Rivas F, Sánchez-Martínez G. Effects of COVID-19 lockdown and a subsequent retraining period on elite athletes' workload, performance, and autonomic responses: a case series. International Journal of Sports Physiology and Performance. 2021;16(11):1707-11.

18. Pereira LA, Nakamura FY, Castilho C, Kitamura K, Kobal R, Abad CCC, et al. The impact of detraining on cardiac autonomic function and specific endurance and muscle power performances of high-level endurance runners. The Journal of sports medicine and physical fitness. 2016;56(12):1583-91.
19. Sarto F, Impellizzeri F, Spörri J, Porcelli S, Olmo J, Requena B, et al. Impact of potential physiological changes due to COVID-19 home confinement on athlete health protection in elite sports: a call for awareness in sports programming. Sports medicine. 2020;50:1417-9.
20. Ross R, Irvin L, Severin R, Ellis B. Return-to-play considerations after COVID-19 infection in elite athletes. Journal of athletic training. 2021;56(10):1061-3.
21. Spyrou K, Alcaraz PE, Marín-Cascales E, Herrero-Carrasco R, Cohen DD, Calleja-Gonzalez J, et al. Effects of the COVID-19 lockdown on neuromuscular performance and body composition in elite futsal players. The Journal of Strength & Conditioning Research. 2021;35(8):2309-15.
22. Melchiorri G, Viero V, Lentini D, Annino G, Tancredi V, Triossi T. Effects of long-term detraining on muscle performance in young soccer players. The Journal of sports medicine and physical fitness. 2023;63(4):521-7.
23. Shaffer F, Ginsberg JP. An overview of heart rate variability metrics and norms. Frontiers in public health. 2017;5:258.
24. Tian Y, He Z-h, Zhao J-x, Tao D-l, Xu K-y, Earnest CP, et al. Heart rate variability threshold values for early-warning nonfunctional overreaching in elite female wrestlers. The Journal of strength & conditioning research. 2013;27(6):1511-9.
25. Marković M, Kukić F, Dopsaj M, Kasum G, Toskic L, Zaric I. Validity of a novel specific wrestling fitness test. The Journal of Strength & Conditioning Research. 2021;35:S51-S7.
26. Liao Y-H, Sung Y-C, Chou C-C, Chen C-Y. Eight-week training cessation suppresses physiological stress but rapidly impairs health metabolic profiles and aerobic capacity in elite taekwondo athletes. PloS one. 2016;11(7):e0160167.
27. Barbieri A, Fuk A, Gallo G, Gotti D, Meloni A, La Torre A, et al. Cardiorespiratory and metabolic consequences of detraining in endurance athletes. Frontiers in Physiology. 2024;14:1334766.
28. Del Vecchio FB, Coswig VS, Cabistany LD, Orcy RB, Gentil P. Effects of exercise cessation on adipose tissue physiological markers related to fat regain: A systematic review. SAGE Open Medicine. 2020;8:2050312120936956.
29. Yoon J. Physiological profiles of elite senior wrestlers. Sports Medicine. 2002;32:225-33.
30. Francino L, Villarroel B, Valdés-Badilla P, Ramirez-Campillo R, Báez-San Martín E, Ojeda-Aravena A, et al. Effect of a six week in-season training program on wrestling-specific competitive performance. International Journal of Environmental Research and Public Health. 2022;19(15):9325.
31. Jeffreys I, Moody J. Strength and Conditioning for Sports Performance. London: Routledge; 2021.
32. Cucinotta D, Vanelli M. WHO declares COVID-19 a pandemic. Acta bio medica: Atenei parmensis. 2020;91(1):157.
33. IOC. Joint statement from the international olympic committee and the Tokyo 2020 organising committee. Olympic news https://www olympic org/news/joint-statement-fromthe-internationalolympic-committee-and-the-tokyo-2020-organising-committee Accessed March. 2020;27:2020.
34. Mujika I, Padilla S. Detraining: loss of training-induced physiological and performance adaptations. Part I: short term insufficient training stimulus. Sports medicine. 2000;30:79-87.

35. Grazioli R, Loturco I, Baroni BM, Oliveira GS, Saciura V, Vanoni E, et al. Coronavirus disease-19 quarantine is more detrimental than traditional off-season on physical conditioning of professional soccer players. The Journal of Strength & Conditioning Research. 2020;34(12):3316-20.
36. Impellizzeri FM, Franchi MV, Sarto F, Meyer T, Coutts AJ. Sharing information is probably more helpful than providing generic training recommendations on return to play after COVID-19 home confinement. Taylor & Francis; 2020. p. 169-70.
37. Casais-Martinez L, Sanroman-Cortes Z, Del Hoyo-Lora M, Solla-Aguiar J, Lago-Penas C, Russian Premier League R. Return to training and competition after COVID-19 in professional football. Science Performance and Science Reports. 2020;1.
38. Melki H, Bouzid MS, Fadhloun M. Correlation between Morphological and Functional Variables during a Specific Wrestling Test for Tunisian Cadet Greco-Roman Wrestlers. Journal of Physical Education and Sport. 2019;19:1282-7.
39. Mohr M, Nassis GP, Brito J, Randers MB, Castagna C, Parnell D, et al. Return to elite football after the COVID-19 lockdown. Managing Sport and Leisure. 2022;27(1-2):172-80.
40. Pichot V, Busso T, Roche F, Garet M, Costes F, Duverney D, et al. Autonomic adaptations to intensive and overload training periods: a laboratory study. Medicine & science in sports & exercise. 2002;34(10):1660-6.
41. Ruiz-Navarro JJ, Plaza-Florido A, Alcantara JM, Gay A, Arellano R. Detraining Effect on Cardiac Autonomic Response to an All-Out Sprint Exercise in Trained Adolescent Swimmers. International Journal of Sports Physiology and Performance. 2023;18(6):573-8.
42. Buchheit M. Monitoring training status with HR measures: do all roads lead to Rome? Frontiers in physiology. 2014;5:73.
43. Gamelin F-X, Berthoin S, Sayah H, Libersa C, Bosquet L. Effect of training and detraining on heart rate variability in healthy young men. International journal of sports medicine. 2007;28(07):564-70.
44. Lagos L, Vaschillo E, Vaschillo B, Lehrer P, Bates M, Pandina R. Heart rate variability biofeedback as a strategy for dealing with competitive anxiety: A case study. Biofeedback. 2008;36(3):109.
45. García-Gonzálvez S, López-Plaza D, Abellán-Aynés O, editors. Influence of competition on anxiety and heart rate variability in young tennis players. Healthcare; 2022: MDPI.
46. Rosa BV, Maldonado AJ, de Araujo AO, Neves LM, Rossi FE, editors. Impact of the COVID-19 Lockdown on the Body Composition and Physical Performance of Athletes: A Systematic Review with Meta-Analysis and Meta-Regression. Healthcare; 2023: MDPI.

Figure 1. Trial Design

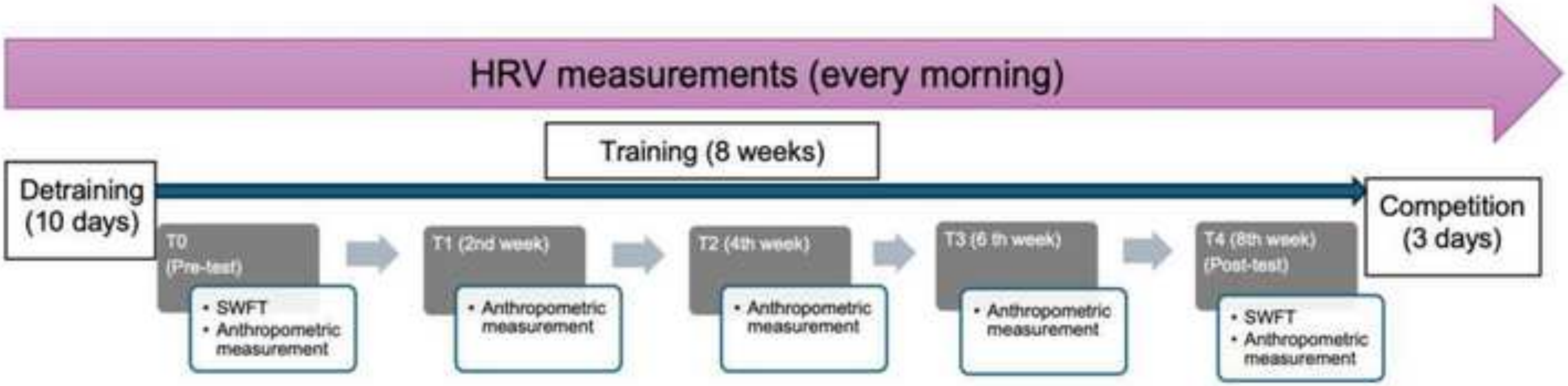

*HRV measured during the detraining for 10 days.
*Pre-tests were measured at the first day of training period
*HRV was measured every morning for 10 days in detraining period, eight weeks during the training period and 3 days during competition period.
*Post-tests were at the end of the T4 period, two days before competition.

Figure 2

Click here to access/download;Figure;figure 2-1.tif

Figure 2. Changes in subcutaneous fat thickness and muscle mass during the non-training and training period.

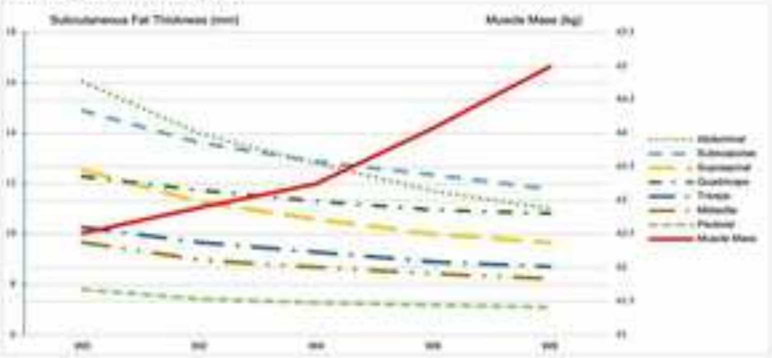

Data in the figure; presented as subcutaneous fat thickness of seven sites and muscle mass. W0: pre-training period; W2: 2 weeks post-training, period; W4: 4 weeks post-training period; W6: 6 weeks post-training period; W8: 8 weeks post-training period

 Click here to access/download;Figure;figure 3.tif

Figure 3. The HRV values during training periods with different loading intensities

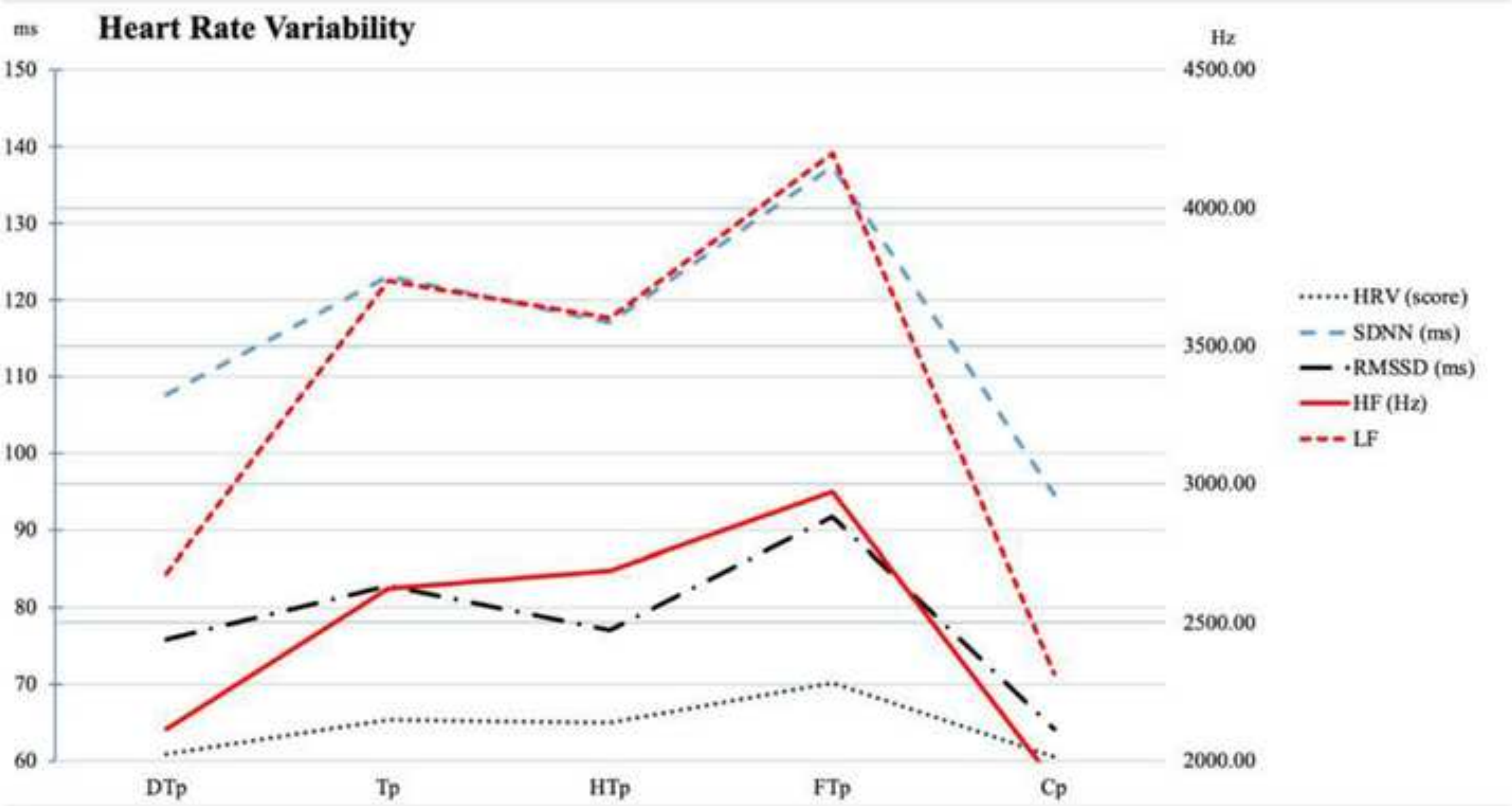

DTp :detraining period; Tp : training period ; HTp: high intensity training period; FTp: form training period ; Cp:competition period; HRV: heart rate variable score; SDNN: standard deviation of NN intervals, RMSSD: root mean square of successive RR interval differences, HF: peak frequency of the high-frequency band, LF: peak frequency of the low-frequency band.

Click here to access/download
Supporting Information
Supplementary Figure-1.tif

Ethical Approval in Turkish

Click here to access/download
Other
ethical approval.jpeg.pdf

Translated Ethical Approval

Click here to access/download

Other

ETHIC ENG.pdf

The impact of 8-week re-training following a 14-week period of training cessation on Greco-Roman Wrestlers

Burhan DEMİRKIRAN[1,2], Ali IŞIN[1,3], Yılmaz SUNGUR[1,4], Tuba MELEKOĞU [1,5*]

[1] Department of Coaching Education, Faculty of Sport Sciences, Akdeniz University, Antalya, Turkey
[2] 0000-0001-7447-627X (ORCID)
[3] 0000-0003-4666-2117 (ORCID)
[4] 0000-0002-0833-051X (ORCID)
[5] 0000-0001-9223-8849 (ORCID)

*Corresponding author: Tuba Melekoğlu, Akdeniz University, Faculty of Sports Sciences, 07070, Antalya/Turkey. phone: +90 242 53196828 e-mail: tmelekoglu@akdeniz.edu.tr

Declarations

Content for publication

Not applicable

Competing interests

The authors declare that they have no competing interests.

Funding

This work was supported by The Scientific Research Projects Coordination Unit of Akdeniz University. Project Number: TYL-2021-5677

Authors' contributions

TM and BD: conceptualization, methodology & data curation; TM: funding acquisition; BD, AI, YS: investigation; TM: supervision; TM, BD, AI, YS: writing - original draft; AI, TM: review & editing.

Acknowledgements

The authors would like to thank the participants for volunteering their time and the Türkiye National Greco-Roman Wrestling Team.

Data availability statement

The data are publicly available at:

https://akdeniz-my.sharepoint.com/:x:/g/personal/tmelekoglu_akdeniz_edu_tr/ERw7tnPaEFlPijwn5fNHUm0B3FSGgQio-tw2uubqm19BgA?e=lYhzkK

https://akdeniz-my.sharepoint.com/:x:/g/personal/tmelekoglu_akdeniz_edu_tr/EZKbAa0dMTlMlyZUE54SUDEBFoduS7qR5dytS-6ukSmnbA?e=oo8oVW

Abstract

Background This study aimed to examine the changes in physical and physiological conditions in elite wrestlers from the Turkish National Wrestling Team, who experienced 14 weeks of restricted physical activity during the COVID-19 lockdown, followed by an 8-week period of retraining and competition.

Methods Twenty male elite wrestlers from the National Greco-Roman Wrestling Team participated in the research. Heart Rate Variability values were measured during the training cessation period and for 8 weeks of subsequent training and then interpreted for training periods with different workloads. Body fat percentage values, initially measured during training cessation, were recorded at 2-week intervals during the training period. To determine the fitness status of wrestlers, the Specific Wrestling Fitness Test was used before and following the 8 weeks of training period.

Results A gradual decrease in both body fat percentage and weight was observed throughout the course of the training period. The SWFT scores showed significant improvements (31.40 ±2.91 vs. 37.40 ±3.22) following the training period. Heart rate variability decreased during the detraining period, progressively improved throughout the 8-week retraining, and subsequently declined during the competition phase, reaching levels similar to those observed during training cessation.

Conclusions Our results suggest that athletes undergo identical reactions in their autonomous nervous system during both competition and training cessation period. Obtaining a comprehensive understanding of these changes can enable coaches and athletes to make accurate decisions in order to optimize training adaptations and attain overall athletic success. Furthermore, over a period of eight weeks following a long non-training period, significant improvements in athletes' body fat,

muscle mass and wrestling performance can be achieved along with training. Moreover, HRV monitoring revealed that autonomic nervous system balance was compromised during both the 14-week detraining and the subsequent competition phases, underlining the need for careful training load management to optimize recovery and performance readiness.

Keywords: Wrestling, training, training cessation, performance, heart rate variability.

Introduction

Elite athletes engage in continuous, high-intensity training that is planned and systematic. This approach is essential for achieving success in international competitions, sustaining achievements, and maintaining peak performance over extended periods. As a result of these high-intensity training regimens, the physiological adaptations observed in elite athletes differ significantly from those seen in sedentary individuals and less-trained athletes. Therefore, athletes who aim to perform at the highest level not only seek to further develop these adaptations but also strive to maintain them. To preserve or enhance these physiological adaptations, it is essential to apply training stimuli at a consistent and adequate level. While excessive training stimuli may lead to overtraining, insufficient or completely discontinued training can result in performance declines known as detraining [1, 2].

High-level competitive athletes who wish to avoid performance loss typically minimize the duration of detraining by limiting breaks from training. However, in certain circumstances—such as injury, chronic conditions, or pregnancy in female athletes— athletes may be forced to stop training. During periods of partial or complete training interruption, the body's physiological responsiveness to exercise stimuli may become diminished or delayed, leading to a slower return to previous performance levels upon retraining [3]. Athletes facing training interruptions may encounter risk factors that negatively impact performance, including declines in muscle strength and size, cardiovascular endurance, along with impairments in neuromuscular coordination and flexibility [3-5]. Additionally, prolonged periods of inactivity are associated with an elevated risk of musculoskeletal injuries upon return to training [6, 7]. When the interruption period lasts for approximately four weeks, reductions are primarily observed in general endurance and muscular

endurance performance, while muscle strength remains largely unaffected [8, 9]. However, if training cessation exceeds four weeks, both muscle strength and endurance show significant declines [3]. Furthermore, reductions in muscle strength and size are influenced by the duration of the detraining period. Short-term detraining typically leads to minimal or no measurable change, whereas extended detraining results in a more rapid loss of muscle size compared to maximal strength [10-13]. Research indicates that after a period of training cessation, skinfold thickness, body fat mass, and percentage tend to increase, while fat-free mass and athletic performance decrease [5].

In elite wrestling, multiple performance components are required to execute consecutive high-intensity physical efforts. Flexibility, agility, reaction time, and the execution of wrestling-specific techniques form the foundation of these components. These attributes are essential for the effective application of strength during performance [14]. Following a 10-week training break in wrestlers, concentric and eccentric isokinetic strengths of the knee and shoulder flexor and extensor were assessed at various angular velocities. Significant decreases in isokinetic muscle strength were detected at angular velocities of 60°, 180°, and 300° [6]. Such reductions not only impair performance but also increase injury risk. In another study, a significant reduction in flexibility and agility was observed among collegiate wrestlers aged 18–21 years after a 10-week training cessation, regardless of changes in muscle mass percentage [14].

Several studies have shown that athletes can regain lost performance relatively quickly following short-term interruptions [15, 16]. Moreover, Valenzuela et al. [17] emphasized that inappropriate or abrupt return-to-play protocols may lead to overuse injuries and compromise full recovery, underscoring the importance of gradual and individualized training resumption. The literature also suggests that muscle strength can be restored rapidly after short-term (~three-week) periods of inactivity. Yasuda, Loenneke (16) reported that individuals with prior strength training experience

were able to regain muscular strength through blood flow restriction training after a three-week detraining phase.

In addition to physical performance losses such as reduced strength and endurance, the autonomic nervous system (ANS)—a vital marker of general health and a determinant of athletic performance—may also be adversely affected by training cessation. Heart rate variability (HRV), which is frequently used as a non-invasive marker of ANS function, can exhibit significant deterioration following prolonged periods of inactivity. Sustained training interruptions are commonly associated with decreased HRV values, which may contribute to reductions in performance capacity [18].

In early 2020, the global outbreak of coronavirus disease (COVID-19) was declared a public health emergency of international concern. In response, social gatherings were restricted and training facilities were closed, causing widespread disruptions in athletic preparation. The impact on elite athletes was substantial, with ongoing championships suspended and major international events postponed.

Moreover, most elite athletes were compelled to train alone in isolated and often unsupervised environments, such as their homes. Upon the easing of lockdown restrictions, it was recommended that training programs be carefully structured and that athletes' performance and health parameters be closely monitored to ensure a safe return to competition. This approach helped minimize injury risk and supported a gradual recovery of physical condition [19, 20].

Although several studies have examined the effects of prolonged inactivity on physical and physiological adaptations and performance in elite athletes, there is a lack of research specifically investigating these effects in elite wrestlers. In particular, little is known about the physiological recovery trajectory following 14 weeks of inactivity in this population. Furthermore, the duration

required for elite wrestlers to fully regain optimal performance capacity in preparation for competition remains insufficiently understood [15, 21, 22].

The purpose of this study was to examine the changes in the physical and physiological parameters in elite wrestlers on the Turkish National Wrestling Team who underwent a 14-week period of limited and unstructured activity during the COVID-19 lockdown, followed by an 8-week retraining and competition phase.

Materials and Methods

Experimental Approach to the Problem

This study is a longitudinal investigation that aims to examine the potential alterations in the physical, physiological, and performance metrics of elite senior national wrestlers competing in the Greco-Roman style. These wrestlers had not engaged in field-based training for a minimum of 14 weeks due to the Covid-19 epidemic. The study focuses on their performance outcomes and autonomous system responses following the resumption of field training. The recruitment period for the study was between 15.10.2020 and 15.02.2021.

During the 14-week COVID-19 lockdown period, information regarding the wrestlers' physical activity levels was obtained through self-reports collected by the Turkish National Wrestling Team coaches. Notably, one of the coaches involved in data collection is also listed as an author of this study, which provided close oversight and contributed to the reliability of the self-reported data. The athletes reported that they remained at home in accordance with pandemic restrictions and engaged only in basic home-based exercises, walking, and limited jogging. They did not participate in any structured or externally supervised training programs, and no specific dietary interventions were implemented. In the early phase of the lockdown, the wrestlers were predominantly inactive due to the uncertainty surrounding the duration of the restrictions. As the lockdown persisted, they

gradually began to engage in limited individual exercises. Nevertheless, these activities lacked the structured intensity, volume, frequency, and duration characteristic of systematic athletic training. HRV is a well-established non-invasive indicator of ANS regulation, specifically reflecting the balance between sympathetic and parasympathetic nervous system activities. Higher HRV is generally associated with increased parasympathetic (vagal) modulation and improved cardiovascular adaptability, whereas reduced HRV reflects sympathetic dominance and impaired autonomic flexibility. [23] In this study, HRV was measured to assess the wrestlers' general health and fitness status during the 8-week retraining period following the training cessation period, and also during the competition period that followed. HRV data were collected using a Polar H10 heart rate chest strap sensor.

To evaluate ANS through HRV, the following commonly used parameters were recorded: standard deviation of NN intervals (SDNN), root mean square of successive RR interval differences (RMSSD), peak frequency of the high-frequency band (HF), peak frequency of the low-frequency band (LF), and the LF/HF power ratio. HRV measurements, averaged over at least 10 days during the training cessation period, were obtained through 5-minute recordings taken while lying in bed immediately upon waking, each morning for 8 weeks after the retraining began.

The athletes' body weight, subcutaneous fat thickness, and body fat percentage were assessed on the first day they of training and subsequently at 2-week intervals throughout the retraining period. In addition, the athletes completed the Specific Wrestling Fitness Test (SWFT), a wrestling-specific assessment, on the first day of retraining and again after eight weeks. Blood lactate samples were collected immediately before, immediately after, and four minutes post-test to determine whether the SWFT was performed at the intended physiological intensity.

Participants

This study included 20 male volunteer wrestlers (aged 21.75 ± 3.11 years) who were members of the Turkish National Wrestling Team and had been training for at least 10 years. The wrestlers included in the study were elite-level athletes who were actively competing prior to the training cessation period. While organized competitions and group training sessions were cancelled during the COVID-19 pandemic, these athletes maintained limited, individualized training routines adapted to the imposed restrictions. However, these activities lacked the structured intensity, volume, frequency, and duration typically required for systematic athletic development. Two of the participants had previously won medals at the European and World Championships, and an additional seven athletes had achieved national and international medals in the year preceding the training cessation. Following the cessation period, one wrestler earned a silver medal at the World Championships and a gold medal the following year. Additionally, six athletes secured medals at European and World Championship events in the seasons following the cessation. The exceptional achievements of the participants, both prior to and following the training cessation period, clearly demonstrate their status as highly competitive elite wrestlers on the international stage.

The inclusion criteria for this study were as follows: being 19 years of age or older; being a national-level athlete with international competition experience; having similar lifestyle habits and sporting backgrounds; not taking any medication that could affect cardiac function; and having no known medical conditions that could impair cardiac or ANS function.

The exclusion criteria included: voluntary withdrawal from the study, failure to regularly attend scheduled measurement sessions, or sustaining an injury or a long-term illness during the training period.

All participants underwent a similar experience during the mandatory stay-at-home period, began retraining at the same time, and followed an identical training program throughout the retraining period. The study design and timeline are illustrated in Figure 1.

All participants provided written informed consent prior to participation. The study was conducted in accordance with the principles of the Declaration of Helsinki. This research was approved by the Akdeniz University Clinical Research Ethics Committee (KAEK-758 / 08.10.2020), and formal permission was obtained from the Wrestling Federation of Türkiye for the measurements.

Figure 1. Trial Design (Approximately Here)

Anthropometric assessments

To evaluate the changes in the physiological characteristics of the participants, body weight (BW), body fat percentage (BFP), body muscle mass (BMM) and body mass index (BMI), measurements were assessed on the last day of training cessation period and 2-week intervals during the 8-week retraining period. Measurements were conducted using a bioelectrical impedance analysis device (Tanita Body Composition Analyzer Type SC-330).

BW, subcutaneous fat thickness (SFT), BFP measurements were measured immediately after training cessation, on the first day of the retraining, and at 2-week intervals thereafter. BMI was calculated using the formula of BMI = Weight (kg)/Height^2 (m^2). BMI values were reported for descriptive purposes only and were complemented with direct body composition measurements such as subcutaneous fat thickness (SFT) to better reflect the athletes' physical profiles. Height, weight, BMI, BFP values were recorded using the integrated printer on the body composition analyzer.

Training program: Due to the constraints imposed by the pandemic, the wrestlers unable to perform a structured training program during the long-term training cessation period. In accordance with the limitations of the COVID-19 conditions, training during this time consisted of basic strength exercises, individual running and walking routines and household-item assisted workouts

that athletes could conduct at home. This period included only self-directed exercise, and no training program supervised by external trainers was implemented.

The retraining period consisted of standardized wrestling training sessions designed and monitored by the national team coaches, conducted at varying intensities over 8 weeks. Following the training cessation period (TCp), training intensity was progressively increased during the first 5 weeks (training period (Tp)). In the 6th and 7th week, training reached its peak (high intensity training period (HTp)). In the 8th week, leading into competition, training intensity was reduced to allow for tapering and recovery (form training period (FTp). After the retraining period, wrestlers participated in an international competition (competition period (Cp)).

Daily training logs were maintained to monitor compliance, and these records were regularly reviewed by the researcher (B.D.). Wrestlers were required to attend at least 90 % of the training sessions. The weekly breakdown of the 8-week training program is provided in Table 1.

Table 1. The weekly content of the 8-week training program

Week 1	Week 2	Week 3	Week 4
**Total exercise session:** 8<br>**Exercise types:**<br>**Type 1-)** 3 BST sessions (%60-70 of 1RM)<br><br>**Type 2-)** 3 WFT (1,5 hours average of each)<br><br>**Type 3-)** 2 AE sessions (1 hour running, %50-60 of HRmax, sauna)	**Total exercise session:** 10<br>**Exercise types:**<br>**Type 1-)** 4 BST sessions (%60-75 of 1RM)<br><br>**Type 2-)** 4 WFT sessions (1,5 hours average of each)<br><br>**Type 3-)** 2 AE (1 hour running, %60-70 of HRmax, sauna)	**Total exercise session:** 11<br>**Exercise types:**<br>**Type 1-)** 4 BST sessions (%70- 75 of 1RM)<br><br>**Type 2-)** 5 WFT sessions (2 hours average of each)<br><br>**Type 3-)** 1 AE session (45 minutes running, %70-75 of HRmax, sauna)	**Total exercise session:** 11<br>**Exercise types:**<br>**Type 1-)** 3 BST sessions (%70- 75 of 1RM)<br><br>**Type 2-)** 6 WFT sessions (2 hours average of each)<br><br>**Type 3-)** 1 HIIT running session (30 minutes, %70-80 of HRmax)

		**Type 4-)** 1 session core exercises	**Type 4-)** 1 session core exercises

Week 5	Week 6	Week 7	Week 8
**Total exercise session:** 10<br>**Exercise types:**<br>**Type 1-)** 3 BST sessions (%75 of 1RM)<br><br>**Type 2-)** 5 WFT sessions (2 hours average of each)<br><br>**Type 3-)** 1 AE session (1 hour running, %60-70 of HRmax, sauna)<br><br>**Type 4-)** 1 session core exercises	**Total exercise session:** 10<br>**Exercise types:**<br>**Type 1-)** 3 SMT session (%50-60 of 1RM)<br>**Type 2-)** 5 WFT sessions (1,5 hours average of each)<br><br>**Type 3-)** 1 HIIT running session (30 minutes, %70-75 of HRmax, sauna)<br><br>**Type 4-)** 1 HIIT running session (20 minutes, 80-90 of HRmax)	**Total exercise session:** 8<br>**Exercise types:**<br>**Type 1-)** 2 EST session (%80-90 of 1RM)<br><br>**Type 2-)** 5 WFT sessions (1 hour average of each)<br><br>**Type 3-)** 1 HIIT running session (20 minutes, 80-90 of HRmax, restitution, sauna)	**Total exercise session:** 6<br>**Exercise types:**<br>**Type 1-)** 2 EST session (%90-100 of 1RM)<br><br>**Type 2-)** 3 WFT sessions (30-45 minutes average of each)<br><br>**Type 3-)** 1 restitution trainings (30 minutes, sauna)

BST: Basic strength training, WFT: Wrestling field training, AE: Aerobic exercise, SMT: Strength maintenance training, HIIT: High intensity interval training, EST: Explosive strength training, HRmax: Maximum heart rate, 1RM: 1 Repetition maximum,

HRV measurements:

HRV was measured daily throughout the training cessation phase, which lasted 10 days prior to the start of the retraining sessions. This phase followed an 8-week training program and a 4-day competition period. Participants were provided a Polar H10 heart rate chest strap sensor (Polar Electro Oy, Kempele, Finland) with a sampling rate of 1000 Hz. A mobile application, Elite HRV, was used to record, store, and export the data.

Prior to data collection, participants were instructed on how to use their assigned HR monitor and smartphone application (Elite HRV). They were directed to measure their resting heart rate for 5 minutes each morning upon waking, while remaining in a supine position. The measurements involved evaluating the R-R intervals, with statistical analyses including SDNN and RMSSD, as well as frequency-domain analyses such as LF, HF, and LF/HF ratio [24]. Mean HRV values were calculated separately for each training phase (TCp, Tp, HTp, FTp, Cp), categorized based on training intensity.

Specific Wrestling Fitness Test (SWFT):

The SWFT was used to evaluate physical fitness capacity of the wrestlers. Participants performed the test before and after the 8-week retraining period. Blood lactate concentrations were measured before, immediately after, and 4 minutes after the SWFT using fingertip samples and a portable lactate analyzer (EDGE Blood Lactate Analyzer, Woodley Equipment Company, Lancashire).

The SWFT consists of maximum wrestling dummy throws within a timed protocol. As wrestling is a weight-classified sport, participants used dummies corresponding to their weight class:

- 22 kg dummy for athletes weighing up to 74.9 kg
- 27 kg dummy for those weighing between 75.0–89.9 kg

Each participant performed three sets of 30-second dummy throws, separated by 20-second rest intervals. After the start signal, participants attempted to throw the dummy as many times as possible within the allotted time using maximum effort [25].

The total number of throws completed across the three sets was recorded as the final test score. Prior to each test, athletes completed a 15-minute general warm-up, followed by a 5-minute specific warm-up involving wrestling dummy throws. A visual illustration of the dummy used in the SWFT is provided in Supplementary Figure 1.

Statistical analyses:

Several statistical methods were employed to test for normality, including assessments of kurtosis and skewness, histogram visualizations, Q-Q plots, and boxplots, followed by the Shapiro-Wilk test.

For comparing pre- and post-test values within groups, either the Dependent Samples t-test or the Wilcoxon signed-rank test was used, depending on data distribution.

To analyze repeated measurements over time, Repeated Measures ANOVA was conducted.

All analyses were performed using Jamovi software (version 2.3.18).

Results

Anthropometric assessments were conducted biweekly throughout the training period. Subcutaneous fat thickness was measured at the triceps, abdomen, subscapular, supraspinale, pectoral, midaxillary, and quadriceps regions using the skinfold method. Additionally, body fat percentage and body muscle mass were assessed using the bioelectrical impedance analysis (BIA) method. Statistically significant improvements were observed in both BFP and BMM parameters at each subsequent measurement point compared to the previous one. (Table 2).

Table 2. Biweekly Changes in Body Weight, Body Fat Percentage, Muscle Mass, and BMI During the Retraining Period

n = 20	W0	W2	W4	W6	W8	F	p	η2
**BW** (kg)	76.49±12.27<br>70;82	75.29±11.58*<br>70;81	74.9±11.33<br>70;80	75.08±11.37<br>70;80	75.47±11.61<br>70;81	6.918	0.000	.27
**BMI** (kg/m²)	25.11±2.74<br>24;26	24.73 2.56**<br>24;26	24.60±2.48<br>23;26	24.67±2.46<br>24;26	24.78±2.44<br>24;26	7.237	0.000	.28
**BFP** (%)	12.38±4.50<br>10;15	11.25±4.19***<br>9;13	10.77±3.96***<br>9;13	10.12±3.77***<br>8;12	9.66±3.66**<br>8;11	44.751	0.000	.70
**BMM** (kg)	62.51±7.86<br>59;66	62.90±7.95**<br>59;67	63.25±8.11***<br>59;67	64.08±8.37***<br>60;68	65.00±8.80***<br>61;69	39.678	0.000	.68

Data in the table; presented as mean ± standard deviation (Mean ± SD) and 95% confidence interval (5%; 95% CI). *p < 0.05, **p < 0.01 ***p < 0.001 compared to the previous measurement. Statistical analysis was performed using repeated measures ANOVA followed by Bonferroni post-hoc tests. W0: pre-training period; W2: 2 weeks post-training, period; W4: 4 weeks post-training period; W6: 6 weeks post-training period; W8: 8 weeks post-training period; BW: body weight; BMI: body mass index; BFP: body fat percentage; BMM: body muscle mass; η2: partial eta squared (effect size).

There was a significant time x group interaction effect in subcutaneous fat thickness measurements. Notable reductions were observed across all assessed sites during the 8-week retraining period (Figure 2). Triceps skinfold thickness decreased significantly at each measurement point compared to the previous one ($p < 0.01$). Similarly, abdominal ($p < 0.001$), supraspinale ($p < 0.001$), midaxillary ($p < 0.01$), subscapular ($p < 0.001$), and quadriceps ($p < 0.01$) regions exhibited statistically significant reductions at every time point. In the pectoral region, a significant reduction

was observed at week 2 ($p < 0.01$) and week 4 ($p < 0.05$); however, no further significant changes were observed thereafter. These results collectively suggest a progressive improvement in body composition during the retraining phase, as evidenced by the consistent decrease in subcutaneous fat thickness across the majority of the measured anatomical sites.

Figure 2. Changes in subcutaneous fat thickness and muscle mass (Approximately here)

SWFT scores increased significantly following the 8-week training period (Table 3.). Despite the improvement in performance, post-test blood lactate levels were lower compared to pre-training values, indicating enhanced anaerobic efficiency and lactate clearance capacity. (Figure 3.).

Table 3. SWFT scores and Lactate Levels

(n = 20)	T0	T4	t	p	E
**SWFT (throws)**	31.40 ±2.91<br>30;33	37.40 ±3.22<br>36;39	10.104	0.000	2.23
			**F**	**p**	**η2**
**LApre (mmol/L)**	1.18 ± 0.33<br>1;1	1.03 ± 0.29<br>1;1	724.028	0.000	0.950
**LApost (mmol/L)**	5.21 ± 2.15<br>4;6	4.88 ± 1.55<br>4;6			
**LA4post (mmol/L)**	10.45 ± 2.84<br>9;12	8.10 ± 1.55<br>7;9			

Data in the table; presented as mean ± standard deviation (Mean ± SD) and 95% confidence interval (5%; 95% CI).
W0: pre-training period; W2: 2 weeks post-training, period; W4: 4 weeks post-training period; W6: 6 weeks post-training period; W8: 8 weeks post-training period; SWFT: specific wrestling fitness test; LApre: Lactate levels pre SWFT; LApost: Lactate levels immediately post SWFT; LA4post: Lactate levels post 4 minutes SWFT; E: effect size, η2: partial eta squared.

Table 4 and Figure 3 present the HRV variables of the participants. A significant time x group interaction was observed for HRV scores, SDNN, RMSSD, LF, LF/HF values. HRV, SDNN, LF values were significantly higher during the FTp compared to both TCp and Cp.

Table 4. HRV Values

(n = 20)	DTp	Tp	HTp	FTp	Cp	F	p	η2
**HRV**	60.80 ± 8.83<br>55;66	65.37 ±6.38<br>62;69	64.97 ± 4.63<br>62;68	70.12 ± 8.91<br>65;76	60.45 ± 6.94<br>56;65	11.384	0.002	0.487

**SDNN**	110.01 ± 55.03<br>77;143	122.76 ± 35.50<br>101;144	116.35 ± 35.02<br>95;138	131.43 ± 37.19<br>109;154	94.60 ± 25.63<br>79;110	5.330	0.001	0.308
**RMSSD**	73.49 ± 40.63<br>49;98	80.66 ± 33.85<br>60;101	75.22 ± 21.86<br>62;88	86.98 ± 27.05<br>71;103	64.06 ± 14.91<br>55;73	2.719	0.040	0.185
**LF**	2456 ± 1049<br>1821;3090	3745 ± 1638<br>2755;4735	3941±1818<br>2392;4590	3979±1775<br>2906;5052	2314±1137<br>1626;3001	6.191	0.000	0.340
**HF**	2172 ± 1161<br>1471;2874	2732 ± 1487<br>1833;3630	2757 ± 1804<br>1667;3848	2946 ± 1583<br>1989;3901	2923 ± 1691<br>907;2939	2.190	0.130	0.154
**LF/HF**	2.65 ± 0.86<br>2;3	4.04 ± 2.11<br>3;5	3.79 ± 1.71<br>3;5	4.08 ± 2.18<br>3;5	2.79 ± 1.24<br>2;4	3.162	0.022	0.209

Data in the table; presented as mean ± standard deviation (Mean ± SD) and 95% confidence interval (5%; 95% CI). DTp :detraining period; Tp : training period ; HTp: high intensity training period; FTp: form training period ; Cp:competition period; HRV: heart rate variable score; SDNN: standard deviation of NN intervals, RMSSD: root mean square of successive RR interval differences, HF: peak frequency of the high-frequency band, LF: peak frequency of the low-frequency band (), and LF/HF: ratio of LF-to-HF power, $\eta 2$: partial eta squared (effect size).

Figure 3. HRV values (Approximately here)

Discussion

This study aimed to investigate the effects of an 8-week retraining program on various physical, physiological, and athletic performance parameters, as well as on ANS tone—specifically examining the balance between the sympathetic and parasympathetic branches—in elite national team wrestlers following a 14-week period of training cessation. The findings revealed significant improvements in body composition, athletic performance, and HRV values—an established indicator of ANS function—following the 8-week retraining period.

Training cessation, or detraining, refers to the partial or complete loss of physiological adaptations previously gained through training, occurring when physical activity is reduced or stopped [3]. Short-term detraining has been reported to result in marked increases in body fat mass (+21%) and reductions in muscle mass (~4%) in elite athletes, potentially impairing performance and agility [26]. When detraining extends beyond four weeks, its negative impact on body fat percentage, muscle mass, and overall performance becomes more pronounced. If prolonged further, it may lead

to the partial or total loss of training-induced adaptations. The magnitude of these changes varies depending on the individual's initial fitness level and the duration of the detraining period. [27]. Previous studies have shown that body fat mass, body fat percentage, and skinfold thickness tend to increase, while fat-free mass and athletic performance decrease following a period of training cessation [5, 28]. In wrestling, maintaining optimal body composition—characterized by a low body fat percentage—is particularly crucial, as athletes are matched against opponents of similar body weight before each bout. A lower fat mass (FM) percentage is considered advantageous, while higher fat-free mass (FFM) is recognized as a key determinant of anaerobic performance in wrestlers. Therefore, balancing fat reduction and muscle preservation is essential for optimizing competitive readiness and athletic capability [29].

Although elite athletes may experience short-term benefits, such as super-compensation and improved recovery following a temporary reduction in training, the long-term consequences of training cessation are detrimental to their overall conditioning and performance. Elite wrestlers typically engage in three to six hours of intensive training per day to optimize their athletic capabilities. As such, their training programs must consistently target the development of strength, flexibility, and anaerobic power to maintain and enhance performance levels. [30, 31]. Under normal conditions, wrestlers take short breaks or reduce training volume and intensity for no more than three weeks after the competitive season. However, the COVID-19 pandemic forced elite athletes into an unusually prolonged period of inactivity. The pandemic led to widespread disruptions in training, limiting access to facilities and structured programs, and resulting in extended periods of detraining that negatively affected physical performance and increased the risk of injury [32, 33]. The adverse effects of prolonged training cessation include impairments in their physical condition and athletic performance, increases in body fat percentage, heightened fatigue,

a greater risk of injury, and longer recovery times [19, 20]. Even inactivity periods as short as 2–4 weeks can induce significant detraining effects, such as reductions in maximal oxygen uptake, muscle strength, neuromuscular coordination, and ventilatory efficiency. These physiological declines can increase susceptibility to injury upon return if training intensity is ramped up too quickly [3, 34].

Grazioli, Loturco (35) reported that an 8-week quarantine period negatively impacted football players' physical performance -specifically power, sprinting, body mass, and body fat mass- and emphasized the need for longer recovery timelines than those associated with regular training. Similarly, Valenzuela et al. [17] highlighted that following long-term inactivity such as during the COVID-19 lockdown, athletes require carefully structured retraining programs lasting at least 6–8 weeks to restore physiological and performance capacities.

Regardless of the cause of training interruption, previous studies have shown that the longer the inactivity period, the more pronounced the declines in endurance, strength, and neuromuscular coordination. These findings underscore the necessity of structured and individualized retraining strategies for athletes returning after extended breaks [3]. Sudden increases in training volume and intensity following confinement can overload physiological systems and elevate injury risk. Conversely, appropriate retraining approaches can minimize injury risk, prevent long-term losses in muscle size and strength, and support a more reliable return to optimal performance. Structured, individualized retraining programs are especially critical for elite wrestlers after prolonged inactivity. To mitigate performance declines and safely return to competition, athletes must implement evidence-based strategies, including progressive training loads, baseline physical assessments, and regular monitoring using tools such as HRV and perceived exertion scales. [15, 16, 36, 37].

In our study, we aimed to determine the physical and physiological adaptations that occur following an eight-week retraining program implemented after a prolonged 14-week period of inactivity. During this retraining period, a clear and progressive improvement was observed in body fat percentage, muscle mass, and athletic performance. However, as noted by Grazioli, Loturco (35) there is currently no research in the literature that allows for direct comparison regarding the time required for such improvements. Nonetheless, our findings suggest that elite wrestlers can achieve substantial gains in body composition and athletic performance within just eight weeks of training, even after an unusually extended period of inactivity.

The development of anaerobic endurance is crucial for explosive sports like wrestling. The SWFT is a widely used performance assessment tool to measure wrestlers' anaerobic endurance and overall physical fitness. The total number of wrestling dummy throws serves as an indicator of immediate physical fitness and anaerobic capacity [25]. Anaerobic endurance, maximal strength, and dynamic balance are fundamental components of wrestling performance. The rapid recovery of these parameters following a period of detraining is essential for maintaining explosive movement patterns and technical efficiency during competition. Although there is currently no study specifically examining the combined effects of training cessation and retraining periods, previous research indicates that wrestlers can improve their SWFT score after six weeks of multi-component training [30, 38]. In combat sports such as wrestling, decision-making skills and technical proficiency require practice against live opponents. Following 4–12 weeks of reduced training (~20–40% of normal load), a gradual return involving general physical preparation and progressive specific skill training over an estimated 3–5 weeks has been recommended to minimize injury risks [39].

In our study, it was observed that elite-level wrestlers significantly improved their SWFT scores by the end of the 8-week retraining period. Moreover, although the SWFT scores increased significantly, post-test lactate levels were notably lower during the retraining phase (8.10 ± 1.55 vs. 10.45 ± 2.84 mmol/L). This reduction in lactate levels, despite an increase in the number of throws performed, indicates improvements both lactate threshold and lactate clearance capacity.

Furthermore, a key finding of our study is the relationship between HRV and both training intensity and competition duration. While consistent long-term training can enhance HRV in athletes, excessively intense training may result in significant daily HRV reductions, and lower morning HRV values may reflect a decline in performance capacity. Athletes experiencing overtraining syndrome often exhibit markedly reduced HRV [40]. In addition, training cessation may also lead to a reduction in HRV. According to Ruiz-Navarro et al. [41], after five weeks of training cessation, all vagal-related HRV measures recorded before and after exercise showed a decline, indicating a deterioration in the swimmers' physical condition. The state of the ANS is influenced by accumulated physical fatigue resulting from increased training loads. Therefore, monitoring HRV can serve as an effective tool for assessing an athlete's training status and overall performance and may also be used to help prevent overtraining [40-42]. Individually tailored training load management can maximize athletic performance gains, whereas both excessive and insufficient training loads may result in detraining and accumulated fatigue. HRV monitoring has been recognized as an effective method for evaluating an athlete's physiological status in relation to sports performance [17].

In our study, HRV values were monitored throughout the eight-week retraining period and the subsequent competition phase. The data were evaluated by analyzing average HRV values across specific time intervals, categorized based on training load intensity. Overall, HRV demonstrated a

progressive improvement during the retraining phase but showed a modest decline during periods of high training intensity. Notably, during the tapering phase - when training intensity was reduced- HRV values reached their peak levels. Valenzuela, Rivas (17) suggested that the COVID-19 lockdown could have a detrimental effect on HRV in elite athletes. Similarly, Gamelin et al. [43]. reported that an eight-week period of training cessation may reverse cardiovascular autonomic adaptations previously achieved through training. On a more optimistic note, Valenzuela, Rivas (17) reported that elite athletes tend to achieve full recovery following 6 to 8 weeks of retraining, which aligns with the findings of our study. However, in our study, although HRV values progressively improved during the retraining phase, they declined again during the subsequent competition period—reaching levels comparable to those observed during the initial training cessation. This decline in HRV suggests that athletes may have experienced increased physiological stress and fatigue during competition, potentially impairing autonomic recovery. Several studies [44, 45] have demonstrated that stress and anxiety negatively influence the ANS during competitive periods, resulting in decreased HRV values. Additionally, research on recovery following injury or forced inactivity consistently shows that restoring muscle mass, cardiovascular capacity, and neuromuscular control require progressive retraining over several weeks to return to baseline levels [19]. It has also been reported that lockdown conditions caused measurable declines in aerobic fitness and neuromuscular performance among elite athletes, underscoring the universal vulnerability of this population to detraining effects, regardless of the reason for inactivity [46]. Collectively, these findings highlight the value of HRV monitoring as a non-invasive tool for assessing athletes' physiological responses to stress and anxiety during competition. Such insights can guide the development of more effective training and recovery strategies. Furthermore, implementing interventions aimed at reducing psychological stress may help maintain optimal HRV levels and support improved performance outcomes in competitive settings.

The most significant limitation of this study is the absence of a control group. After the isolation period ended, all athletes were allowed and required to resume training simultaneously. In elite athletic populations, establishing a control group is particularly challenging, as training cessation or alterations to the standard program are generally not accepted by coaches or athletes.

Another limitation is the lack of pre-pandemic or during-pandemic baseline measurements. Due to the sudden and unforeseen implementation of pandemic-related restrictions, initial assessments could not be conducted in accordance with mandatory social distancing regulations. As a result, baseline measurements were taken at the beginning of the retraining phase, and subsequent changes were tracked over time. Additionally, the study employed a convenience sampling method, as access to participants was severely restricted during the COVID-19 pandemic. Although this approach was the most practical under the given circumstances, it may limit the generalizability of the findings. Future studies are encouraged to adopt randomized or stratified sampling methods to enhance sample representativeness and strengthen the robustness of the conclusions.

Elite athletes possess a remarkable capacity to regain both athletic performance and body composition following periods of training cessation. HRV, a key marker of ANS function, fluctuates significantly in response to changes in training load and competition stress. These fluctuations underscore the importance of HRV monitoring for optimizing performance and recovery strategies.

A thorough understanding of these physiological responses can empower coaches and athletes to make more informed decisions, facilitate targeted adaptations, and ultimately achieve higher levels of athletic success. Based on the findings of our study, it is recommended that wrestlers returning from prolonged training cessation follow retraining programs that emphasize anaerobic conditioning, technical-tactical drills performed under fatigue, and gradual progression of training loads to minimize injury risk and accelerate the restoration of competitive readiness.

Conclusions

As a result of eight-week retraining period, a clear and progressive decrease in body fat percentage and an increase in muscle mass were observed. Following this training period, the Specific Wrestling Fitness Test (SWFT), which is used to assess wrestlers' physical fitness capacity, showed significant improvements in performance scores.

Throughout the retraining period, HRV values exhibited a gradual upward trend, reflecting improved autonomic balance. However, a slight decline in HRV was noted during phases of intensive training. During the tapering phase, characterized by reduced training intensity, HRV values reached their highest levels.

These findings indicate that HRV is highly sensitive to variations in training load and competitive stress. Notably, HRV values measured during the competition phase were similar to those observed during the non-training period, suggesting a potential physiological strain associated with competitive demands.

Acknowledgement

The English language, grammar, and overall clarity of this manuscript were reviewed and edited with the assistance of an advanced AI language model (ChatGPT-4o, OpenAI). Following the AI-supported editing, final revisions and adaptations were carefully made by the authors to ensure that the manuscript meets international academic writing standards and accurately reflects the intended scientific meaning.

References

1. MacInnis MJ, Gibala MJ. Physiological adaptations to interval training and the role of exercise intensity. The Journal of physiology. 2017;595(9):2915-30.

2. Schoenfeld BJ, Grgic J, Ogborn D, Krieger JW. Strength and hypertrophy adaptations between low-vs. high-load resistance training: a systematic review and meta-analysis. The Journal of Strength & Conditioning Research. 2017;31(12):3508-23.
3. Mujika I, Padilla S. Detraining: loss of training-induced physiological and performance adaptations. Part II: long term insufficient training stimulus. Sports medicine. 2000;30:145-54.
4. Bosquet L, Berryman N, Dupuy O, Mekary S, Arvisais D, Bherer L, et al. Effect of training cessation on muscular performance: A meta-analysis. Scandinavian journal of medicine & science in sports. 2013;23(3):e140-e9.
5. Alvurdu S, Baykal C, Akyildiz Z, Şenel Ö, Silva AF, Conte D, et al. Impact of prolonged absence of organized training on body composition, neuromuscular performance, and aerobic capacity: A study in youth male soccer players exposed to COVID-19 lockdown. International journal of environmental research and public health. 2022;19(3):1148.
6. Noorbakhsh M, Zarei M, Hovanloo F, Hoseini A, Yaghoubitajani Z. Exploring the influence of a 10-week specific detraining on injury risk factors among elite young wrestlers: a prospective study. Scientific Reports. 2025;15(1):7348.
7. Yamashita D, Hirata K, Yamazaki K, Mujika I, Miyamoto N. Effect of two weeks of training cessation on concentric and eccentric knee muscle strength in highly trained sprinters. Plos one. 2023;18(7):e0288344.
8. Chen Y-T, Hsieh Y-Y, Ho J-Y, Lin T-Y, Lin J-C. Two weeks of detraining reduces cardiopulmonary function and muscular fitness in endurance athletes. European Journal of Sport Science. 2022;22(3):399-406.
9. Pereira LA, Freitas TT, Pivetti B, Alcaraz PE, Jeffreys I, Loturco I. Short-term detraining does not impair strength, speed, and power performance in elite young soccer players. Sports. 2020;8(11):141.
10. Achten J, Jeukendrup AE. Heart rate monitoring: applications and limitations. Sports medicine. 2003;33:517-38.
11. Häkkinen K, Alen M, Kallinen M, Newton R, Kraemer W. Neuromuscular adaptation during prolonged strength training, detraining and re-strength-training in middle-aged and elderly people. European journal of applied physiology. 2000;83:51-62.
12. Räntilä A, Ahtiainen JP, Avela J, Restuccia J, Kidgell D, Häkkinen K. High responders to hypertrophic strength training also tend to lose more muscle mass and strength during detraining than low responders. The Journal of Strength & Conditioning Research. 2021;35(6):1500-11.
13. Halonen EJ, Gabriel I, Kelahaara MM, Ahtiainen JP, Hulmi JJ. Does Taking a Break Matter—Adaptations in Muscle Strength and Size Between Continuous and Periodic Resistance Training. Scandinavian Journal of Medicine & Science in Sports. 2024;34(10):e14739.
14. Hedya MHM. The Effect of A Detraining on some physical variables and body composition during the transition period for wrestlers. Assiut Journal of Sport Science and Arts. 2016;116(1):628-47.
15. Stokes KA, Jones B, Bennett M, Close GL, Gill N, Hull JH, et al. Returning to play after prolonged training restrictions in professional collision sports. International journal of sports medicine. 2020;41(13):895-911.
16. Yasuda T, Loenneke JP, Ogasawara R, Abe T. Effects of short-term detraining following blood flow restricted low-intensity training on muscle size and strength. Clinical physiology and functional imaging. 2015;35(1):71-5.
17. Valenzuela PL, Rivas F, Sánchez-Martínez G. Effects of COVID-19 lockdown and a subsequent retraining period on elite athletes' workload, performance, and autonomic responses: a case series. International Journal of Sports Physiology and Performance. 2021;16(11):1707-11.

18. Pereira LA, Nakamura FY, Castilho C, Kitamura K, Kobal R, Abad CCC, et al. The impact of detraining on cardiac autonomic function and specific endurance and muscle power performances of high-level endurance runners. The Journal of sports medicine and physical fitness. 2016;56(12):1583-91.

19. Sarto F, Impellizzeri F, Spörri J, Porcelli S, Olmo J, Requena B, et al. Impact of potential physiological changes due to COVID-19 home confinement on athlete health protection in elite sports: a call for awareness in sports programming. Sports medicine. 2020;50:1417-9.

20. Ross R, Irvin L, Severin R, Ellis B. Return-to-play considerations after COVID-19 infection in elite athletes. Journal of athletic training. 2021;56(10):1061-3.

21. Spyrou K, Alcaraz PE, Marín-Cascales E, Herrero-Carrasco R, Cohen DD, Calleja-Gonzalez J, et al. Effects of the COVID-19 lockdown on neuromuscular performance and body composition in elite futsal players. The Journal of Strength & Conditioning Research. 2021;35(8):2309-15.

22. Melchiorri G, Viero V, Lentini D, Annino G, Tancredi V, Triossi T. Effects of long-term detraining on muscle performance in young soccer players. The Journal of sports medicine and physical fitness. 2023;63(4):521-7.

23. Shaffer F, Ginsberg JP. An overview of heart rate variability metrics and norms. Frontiers in public health. 2017;5:258.

24. Tian Y, He Z-h, Zhao J-x, Tao D-l, Xu K-y, Earnest CP, et al. Heart rate variability threshold values for early-warning nonfunctional overreaching in elite female wrestlers. The Journal of strength & conditioning research. 2013;27(6):1511-9.

25. Marković M, Kukić F, Dopsaj M, Kasum G, Toskic L, Zaric I. Validity of a novel specific wrestling fitness test. The Journal of Strength & Conditioning Research. 2021;35:S51-S7.

26. Liao Y-H, Sung Y-C, Chou C-C, Chen C-Y. Eight-week training cessation suppresses physiological stress but rapidly impairs health metabolic profiles and aerobic capacity in elite taekwondo athletes. PloS one. 2016;11(7):e0160167.

27. Barbieri A, Fuk A, Gallo G, Gotti D, Meloni A, La Torre A, et al. Cardiorespiratory and metabolic consequences of detraining in endurance athletes. Frontiers in Physiology. 2024;14:1334766.

28. Del Vecchio FB, Coswig VS, Cabistany LD, Orcy RB, Gentil P. Effects of exercise cessation on adipose tissue physiological markers related to fat regain: A systematic review. SAGE Open Medicine. 2020;8:2050312120936956.

29. Yoon J. Physiological profiles of elite senior wrestlers. Sports Medicine. 2002;32:225-33.

30. Francino L, Villarroel B, Valdés-Badilla P, Ramirez-Campillo R, Báez-San Martín E, Ojeda-Aravena A, et al. Effect of a six week in-season training program on wrestling-specific competitive performance. International Journal of Environmental Research and Public Health. 2022;19(15):9325.

31. Jeffreys I, Moody J. Strength and Conditioning for Sports Performance. London: Routledge; 2021.

32. Cucinotta D, Vanelli M. WHO declares COVID-19 a pandemic. Acta bio medica: Atenei parmensis. 2020;91(1):157.

33. IOC. Joint statement from the international olympic committee and the Tokyo 2020 organising committee. Olympic news https://www olympic org/news/joint-statement-fromthe-internationalolympic-committee-and-the-tokyo-2020-organising-committee Accessed March. 2020;27:2020.

34. Mujika I, Padilla S. Detraining: loss of training-induced physiological and performance adaptations. Part I: short term insufficient training stimulus. Sports medicine. 2000;30:79-87.

35. Grazioli R, Loturco I, Baroni BM, Oliveira GS, Saciura V, Vanoni E, et al. Coronavirus disease-19 quarantine is more detrimental than traditional off-season on physical conditioning of professional soccer players. The Journal of Strength & Conditioning Research. 2020;34(12):3316-20.
36. Impellizzeri FM, Franchi MV, Sarto F, Meyer T, Coutts AJ. Sharing information is probably more helpful than providing generic training recommendations on return to play after COVID-19 home confinement. Taylor & Francis; 2020. p. 169-70.
37. Casais-Martinez L, Sanroman-Cortes Z, Del Hoyo-Lora M, Solla-Aguiar J, Lago-Penas C, Russian Premier League R. Return to training and competition after COVID-19 in professional football. Science Performance and Science Reports. 2020;1.
38. Melki H, Bouzid MS, Fadhloun M. Correlation between Morphological and Functional Variables during a Specific Wrestling Test for Tunisian Cadet Greco-Roman Wrestlers. Journal of Physical Education and Sport. 2019;19:1282-7.
39. Mohr M, Nassis GP, Brito J, Randers MB, Castagna C, Parnell D, et al. Return to elite football after the COVID-19 lockdown. Managing Sport and Leisure. 2022;27(1-2):172-80.
40. Pichot V, Busso T, Roche F, Garet M, Costes F, Duverney D, et al. Autonomic adaptations to intensive and overload training periods: a laboratory study. Medicine & science in sports & exercise. 2002;34(10):1660-6.
41. Ruiz-Navarro JJ, Plaza-Florido A, Alcantara JM, Gay A, Arellano R. Detraining Effect on Cardiac Autonomic Response to an All-Out Sprint Exercise in Trained Adolescent Swimmers. International Journal of Sports Physiology and Performance. 2023;18(6):573-8.
42. Buchheit M. Monitoring training status with HR measures: do all roads lead to Rome? Frontiers in physiology. 2014;5:73.
43. Gamelin F-X, Berthoin S, Sayah H, Libersa C, Bosquet L. Effect of training and detraining on heart rate variability in healthy young men. International journal of sports medicine. 2007;28(07):564-70.
44. Lagos L, Vaschillo E, Vaschillo B, Lehrer P, Bates M, Pandina R. Heart rate variability biofeedback as a strategy for dealing with competitive anxiety: A case study. Biofeedback. 2008;36(3):109.
45. García-Gonzálvez S, López-Plaza D, Abellán-Aynés O, editors. Influence of competition on anxiety and heart rate variability in young tennis players. Healthcare; 2022: MDPI.
46. Rosa BV, Maldonado AJ, de Araujo AO, Neves LM, Rossi FE, editors. Impact of the COVID-19 Lockdown on the Body Composition and Physical Performance of Athletes: A Systematic Review with Meta-Analysis and Meta-Regression. Healthcare; 2023: MDPI.

Reviewer #2:

We sincerely thank you for your valuable comments and suggestions. We have carefully addressed each of your points and revised the manuscript accordingly. Below, we provide a detailed, point-by-point response. The authors' responses are written in italics. Where appropriate, changes made in the manuscript have been highlighted in yellow.

Suggestion: Page 18, line 26 – authors failed to report how long the detraining phase was in the abstract section. If you state that retraining period was 8 weeks, it would be of great value to also know how long was the detraining phase.

Response: *Thank you for your valuable comment. As you rightly pointed out, clearly stating the duration of the detraining phase adds clarity and value to the abstract and overall interpretation. Although the 14-week detraining period was already mentioned in the background section in abstract, we have now revised the conclusion section to explicitly include the detraining duration in the relevant sentence for improved clarity. The revised sentence now reads:*

"Moreover, HRV monitoring revealed that autonomic nervous system balance was compromised during both the 14-week detraining and the subsequent competition phases, underlining the need for careful training load management to optimize recovery and performance readiness."

We hope this revision addresses your concern.

Suggestion: Page 19, line 44 – "During these periods of partial or complete training interruption, it has been suggested that the organism's responsiveness to exercise is significantly prolonged". I am not sure what this sentence means. Refine for clarity.

Response: *Thank you for your comment. We agree that the original sentence lacked clarity and could be misinterpreted. To improve the precision of the statement, we have revised the sentence on Page 19, line 44 as follows:*

"During periods of partial or complete training interruption, the body's physiological responsiveness to exercise stimuli may become diminished or delayed, leading to a slower return to previous performance levels upon retraining."

We believe this updated version more accurately reflects the intended meaning and appreciate your helpful suggestion.

Suggestion: Page 19, line 46 – "Athletes facing training interruptions may encounter risk factors that negatively impact performance, including declines in muscle strength, muscle size, cardiovascular endurance, as well as impairments in neuromuscular coordination and flexibility." Provide citation(s).

Response: *We have added relevant citations to the sentence on Page 19, line 46, which now reads:*

"Athletes facing training interruptions may encounter risk factors that negatively impact performance, including declines in muscle strength, muscle size, cardiovascular endurance, as well as impairments in neuromuscular coordination and flexibility (Alvurdu et al., 2022; Bosquet et al., 2013; Mujika & Padilla, 2000b).

Suggestion: Page 21, line 79 – "Proper training methods can minimize the risk of injury, prevent long-term losses in muscle size and strength, and facilitate a faster and more reliable return to optimal fitness and performance levels. Structured and individualized retraining programs are crucial for minimizing injury risk and accelerating performance recovery in elite wrestlers following prolonged periods of training cessation. Given the detrimental effects of training cessation on athletic performance, it is essential for athletes to implement appropriate strategies to mitigate performance declines." Move this section to discussion. It deviates from the key points in the Introduction and it makes too long.

Response: *As recommended, we have moved the relevant section from the Introduction to the Discussion to maintain a clearer focus on the key points and improve the overall structure and flow of the manuscript. We believe this adjustment enhances both the coherence and readability of the paper.*

Suggestion: Page 24, line 148 – which device did you use to measure HRV?

Response: *We have revised the sentence to include the specific device used for HRV measurement. The updated sentence now reads:*

"HRV data were collected using a Polar H10 heart rate chest strap sensor."

Suggestion: Page 24, line 157 – Turkish national team I'd assume? Mention this. It's important.

Response: *We have revised the sentence to clearly indicate that the participants were members of the Turkish National Wrestling Team. The updated sentence now reads:*

"*This study included 20 male volunteer wrestlers (aged 21.75 ± 3.11 years) who were members of the Turkish National Wrestling Team and had been training for at least 10 years."*

Suggestion: Page 26, line 199 – they COULD not… Stay in the past tense.

Response: *As suggested, we have revised the sentence to remain consistent with the past tense. The corrected version now reads:*

"Due to the constraints imposed by the pandemic, the wrestlers unable to perform a structured training program during the long-term training cessation period."

Suggestion: Page 26, line 213 – WERE required. The same principle applies.

Response: *As suggested, we have revised the sentence to remain consistent with the past tense. The corrected version now reads:*

"Wrestlers were required to attend at least 90 % of the training sessions. The weekly breakdown of the 8-week training program is provided in Table 1."

Suggestion: Page 28, line 239 – "The participant was performed three sets".

Response: *As suggested, we have revised the sentence to remain consistent with the past tense. The corrected version now reads:*

"Each participant performed three sets of 30-second dummy throws, separated by 20-second rest intervals."

Suggestion: As I said in the initial review, this manuscript would benefit greatly if the native English-speaking person reads it.

Response: *As per your suggestion, the manuscript has been thoroughly reviewed and revised by a native English speaker to improve its linguistic quality, clarity, and fluency."*
