# Supplementary material for: The impact of 8-week re-training following a 14-week period of training cessation on Greco-Roman Wrestlers
Source: PLoS One. 2025 Jun 25;20(6):e0326731. doi: 10.1371/journal.pone.0326731 (PMC12194023; doi:10.1371/journal.pone.0326731)
Supplement: Attachment — Submitted filename: Responso to revievers.docx [file pone.0326731.s002.docx]

**Reviewer #1:**

We sincerely thank you for your valuable comments and suggestions. We have carefully addressed each of your points and revised the manuscript accordingly. Below, we provide a detailed, point-by-point response. The authors’ responses are written in italics. Where appropriate, changes made in the manuscript have been highlighted in yellow.

**Suggestion:** The problems and risk factors that athletes encounter after the training stop period should be expressed more clearly and in detail.

**Response:** *In the Introduction and Abstract sections, we added information about decreases in muscle strength, cardiovascular endurance, flexibility, neuromuscular coordination, and the increased injury risk associated with training cessation. The following paragraph has been added to the manuscript:*

“Athletes facing training interruptions may encounter risk factors that negatively impact performance, including declines in muscle strength, muscle size, cardiovascular endurance, as well as impairments in neuromuscular coordination and flexibility. Additionally, prolonged periods of inactivity are associated with an elevated risk of musculoskeletal injuries upon return to training”

**Suggestion:** The reason why it is important to use the right method after the training stop period should be expressed more clearly.

**Response:** *The Abstract was revised to emphasize that appropriate retraining methods are critical for minimizing injury risk and for rapidly regaining optimal performance. The following paragraph has been added to the manuscript:*

“Proper training methods can minimize the risk of injury, prevent long-term losses in muscle size and strength, and facilitate a faster and more reliable return to optimal fitness and performance levels. Structured and individualized retraining programs are crucial for minimizing injury risk and accelerating performance recovery in elite wrestlers following prolonged periods of training cessation. Given the detrimental effects of training cessation on athletic performance, it is essential for athletes to implement appropriate strategies to mitigate performance declines. Several studies have indicated that athletes are capable of regaining lost performance relatively quickly after short-term interruptions (Stokes et al., 2020; Yasuda et al., 2015). Moreover, Valenzuela et al. (Valenzuela et al., 2021) emphasized that inappropriate or abrupt return-to-play protocols might lead to overuse injuries and hinder full recovery, highlighting the critical need for gradual and individualized training resumption.”

**Suggestion:** It should be stated which sporting performance parameters the training stop period can affect in wrestling in particular.

**Response:** *Wrestling-specific performance parameters, including muscle strength, flexibility, agility, and reaction time, were detailed in the Abstract and Introduction. The following paragraph has been added to the manuscript:*

“In elite wrestling, multiple performance components are required to perform consecutive high-intensity physical activities. Flexibility, agility, reaction time, and the execution of wrestling-specific techniques form the foundation of these components. These elements are essential for the correct and efficient application of physical strength during performance (Hedya, 2016). Following a 10-week training break in wrestlers, the concentric and eccentric isokinetic strengths of the knee and shoulder flexor and extensor muscles were evaluated at different angular velocities. Significant decreases in isokinetic muscle strength were detected at angular velocities of 60°, 180°, and 300° (Noorbakhsh et al., 2025). Such declines in muscle strength not only contribute to performance losses but also represent a major risk factor for injuries. In another study, a significant reduction in flexibility and agility was observed among collegiate wrestlers aged 18–21 years after a 10-week training cessation, independent of changes in muscle mass percentage (Hedya, 2016).”

**Suggestion:** Since the most striking aspect of the research is the importance of correct loading and quickly reaching optimum performance after a long training stop period, this issue should be emphasized more.

**Response:** *We revised both the Abstract and the Discussion sections to highlight the importance of correct loading strategies in achieving rapid and safe performance recovery.*

In Abstract:

“Obtaining a comprehensive understanding of these changes can enable coaches and athletes to make accurate decisions in order to optimize training adaptations and attain overall athletic success.”

“Furthermore, over a period of eight weeks following a long non-training period, significant improvements in athletes’ body fat, muscle mass and wrestling performance can be achieved along with training.”

In Discussion:

“The proper methods can minimize the risks of injury, prevent long-term loss of performance such as muscle size and strength and allow a faster and more reliable return to optimum fitness and conditions. Structured and individualized retraining programs appear crucial in minimizing the risks of injury and accelerating performance recovery in elite wrestlers after prolonged training cessation.”

**Materials and Methods**

**Suggestion:** More detailed information should be provided about which period the athletes are in before the training stop period (in-season, end of season or rest period).

**Response:** *It was clarified that the athletes were in their in-season period before the onset of the training cessation.*

“The wrestlers in the research group consisted of elite level athletes who were in-season before the training cessation period.”

**Suggestion:** The training program applied to the athletes for 8 weeks should be expressed in more detail.

**Response:** *We provided a detailed description of the 8-week training program and included a week-by-week training content in Table 1.*

**Suggestion:** The movements and their durations preferred in the training program applied to the athletes should be clearly expressed in tables.

**Response:** *A comprehensive table (Table 1) was added, detailing the exercise types, session frequency, intensity, and weekly structure.*

**Suggestion:** It should be stated more clearly how the athletes follow up whether or not they apply the specified training during the 8-week period.

**Response:** *We added information about daily training records being kept and reviewed by a researcher (B.D.), ensuring at least 90% session compliance.*

“Daily training records were kept for the athlete to follow the training, and these records were regularly reviewed by the researcher (B.D.). Wrestlers are required to perform 90 % of the training sessions.”

**Suggestion:** It is recommended to add the visual of the wrestling mannequin used in SWFT to increase the explanatory power of the test used.

**Response**: *In accordance with the recommendation, we have added visuals of the wrestling mannequins used in the Specific Wrestling Fitness Test (SWFT) to enhance the explanatory power and clarity of the method section. The images have been provided as a supplementary file and are referenced appropriately within the manuscript.*

**Results**

**Suggestion:** The statistical methods applied and the analyses included in the article are explanatory and appropriate for the purpose.

**Response:** *No change needed. Statistical analyses were already appropriate, but minor clarifications regarding repeated measures ANOVA and other tests were added.*

**Discussion**

**Suggestion:** Since the main purpose of the research is to ensure fast and accurate recovery after a long period without training, the importance of this subject should be emphasized more.

**Response:** *We emphasized this point in the opening and closing sections of the Discussion.*

“The proper methods can minimize the risks of injury, prevent long-term loss of performance such as muscle size and strength and allow a faster and more reliable return to optimum fitness and conditions. Structured and individualized retraining programs appear crucial in minimizing the risks of injury and accelerating performance recovery in elite wrestlers after prolonged training cessation. Due to the negative impact of training cessation on athletic performance, it is essential for athletes to adopt appropriate strategies to counteract the decline in performance. Indeed, several studies have emphasized that athletes are able to regain lost performance relatively quickly after short-term cessations [1, 2]. Moreover, Valenzuela et al. [3] emphasized that inappropriate or abrupt return-to-play protocols might lead to overuse injuries and hinder full recovery, highlighting the critical need for gradual and individualized training resumption. ”

Recent findings suggest that after a long period of inactivity such as the COVID-19 lockdown, athletes require a carefully structured retraining program lasting at least 6–8 weeks to restore their physiological and performance capacities [3]. Independent of the cause of training cessation, previous research has shown that the longer the inactivity period, the greater the loss in endurance, strength, and neuromuscular coordination, thereby emphasizing the importance of carefully structured retraining strategies for athletes returning after prolonged inactivity [4]. Sudden increases in training volume and intensity after a period of confinement may lead to an overload of physiological systems, raising the risk of injuries. Properly managed progressive loading, baseline physical assessments, and monitoring tools such as heart rate variability and perceived exertion scales are essential to safely reinstate competitive training [5, 6].

**Suggestion:** Although there is no similar research on this subject during the Covid-19 period, a comparison should be made in the discussion section with studies that express how many weeks and which methods are applied to athletes, especially after injuries or when the athlete is away from the field for a long time.

**Response:**  *We discussed and compared findings with studies focused on recovery strategies after injuries and long-term detraining, expanding the context beyond COVID-19.*

Grazioli et al. (2020) reported that an 8-week quarantine period negatively impacted football players' physical performance, including power, sprinting, body mass, and body fat mass, and suggested that athletes needed longer recovery periods than regular training programs. Recent findings suggest that after a long period of inactivity such as the COVID-19 lockdown, athletes require a carefully structured retraining program lasting at least 6–8 weeks to restore their physiological and performance capacities (Valenzuela et al., 2021).

**Suggestion:** Although the relevant research was conducted in a specific process such as Covid-19, the findings obtained are very important for athletes who have to stop training for a long time. Therefore, more space should be given to similar studies suitable for the specified athlete profile.

**Response:** *We included new paragraphs connecting our findings to broader cases (e.g., injury-related absences), discussing the applicability of the results.*

“Several studies [32-34] have shown that stress and anxiety affect the autonomic nervous system in competition period, resulting in a decline in heart rate variability values.”

“It was reported that short-term detraining induces significant increases in body fat mass (+21%) and reductions in muscle mass (~4%) among elite athletes, which may negatively affect performance and agility [7].”

Recent findings suggest that after a long period of inactivity such as the COVID-19 lockdown, athletes require a carefully structured retraining program lasting at least 6–8 weeks to restore their physiological and performance capacities (Valenzuela et al., 2021). Independent of the cause of training cessation, previous research has shown that the longer the inactivity period, the greater the loss in endurance, strength, and neuromuscular coordination, thereby emphasizing the importance of carefully structured retraining strategies for athletes returning after prolonged inactivity (Mujika & Padilla, 2000).

**Suggestion:** In the relevant studies, the importance of the parameters examined for the wrestling branch and how these parameters can affect performance with rapid recovery should be included in the foresight and similar studies. -More suggestions should be made regarding the results of the research, specific to the wrestling branch.

**Response:** *In response, we have expanded the discussion section by emphasizing how the key physiological and performance parameters assessed in our study, such as anaerobic endurance, maximal strength, and dynamic balance, are critical for rapid recovery and optimal performance in wrestling. We have also incorporated references to support the wrestling-specific importance of these parameters. Accordingly, we have added practical recommendations based on our findings, specifically tailored to the needs of wrestlers. These include implementing periodized retraining programs emphasizing anaerobic conditioning, technical drills under fatigue, and injury prevention strategies during return-to-training phases after long-term inactivity.*

“Anaerobic endurance, maximal strength, and dynamic balance are fundamental components for wrestling performance. Rapid recovery of these parameters following a period of detraining is essential for maintaining explosive movement patterns and technical efficiency during competition.”

“Sudden increases in training volume and intensity after a period of confinement may lead to an overload of physiological systems, raising the risk of injuries. Properly managed progressive loading, baseline physical assessments, and monitoring tools such as heart rate variability and perceived exertion scales are essential to safely reinstate competitive training (Casais-Martinez et al., 2020; Impellizzeri et al., 2020). “

“The fact that the lactate level is lower despite the increase in the throws performed in the SWFT test indicates that the lactate threshold and lactate removal capacity were also developed.”

**Reviewer #2:**

We sincerely thank the reviewer for their valuable time, insightful suggestions, and constructive feedback, all of which have significantly contributed to improving the quality and clarity of our manuscript. We carefully considered each comment and made the necessary revisions accordingly. Below, we provide a detailed, point-by-point response. The authors’ responses are written in italics. Where appropriate, changes made in the manuscript have been highlighted in green.

**Suggestion:** Page 12, line 25 – so you have mentioned that HRV is the key variable observed and then you don’t mention the effects of detraining and retraining on it?

**Response:** *We appreciate the reviewer’s insightful comment. We have revised the abstract to explicitly state that heart rate variability (HRV) decreased during the training cessation period, progressively improved during the retraining phase, and subsequently declined again during the competition phase, approaching values observed during the detraining period. This highlights the sensitivity of HRV to training load fluctuations and competitive stress. In addition to revising the main body of the abstract, we have also updated the conclusion section to emphasize that HRV was negatively impacted not only during detraining but also during the competition phase. This addition highlights the practical importance of HRV monitoring for managing training loads and optimizing athlete recovery and readiness for competition.*

“Heart rate variability decreased during the detraining period, progressively improved throughout the 8-week retraining, and subsequently declined during the competition phase, reaching levels similar to those observed during training cessation.”

“Moreover, HRV monitoring revealed that autonomic nervous system balance was compromised during both the detraining and competition phases, underlining the need for careful training load management to optimize recovery and performance readiness.”

**Suggestion:** Line 14, line 62 – you mean the prevention of social gatherings?

**Response:** *In the revised manuscript, we have specified that the COVID-19 lockdown measures involved the prevention of social gatherings, resulting in training disruptions and cessation of organized sports activities. The phrasing has been adjusted accordingly to improve clarity.*

As a result of the pandemic, the prevention of social gatherings and the closure of training facilities caused widespread disruptions in athletic training routines.

**Suggestion:** Page 14, line 69 – why do we assume that athletes were not active during COVID pandemic? They were competing okay, but many of them remained active in the conditions they were given.

**Response:** *We agree that not all athletes were completely inactive during the COVID-19 pandemic. Accordingly, we have revised the relevant sentences to reflect that athletes experienced restricted or limited training opportunities rather than complete inactivity. The revised text clarifies that training activities were curtailed or adapted based on the available conditions rather than being fully suspended.*

*We would also like to clarify that by “physical inactivity,” we specifically refer to wrestlers’ inability to engage in structured, systematic training programs during the pandemic. Although athletes performed individual home-based exercises during lockdowns and social isolation measures, these activities lacked the organized structure typically required in terms of training intensity, volume, frequency, and duration. We have revised the manuscript to incorporate this clarification, aiming to make the study context and purpose more precise and comprehensible.*

The wrestlers included in the study were elite-level athletes who were actively in-season before the training cessation period. While organized competitions and group training sessions were cancelled during the COVID-19 pandemic, these athletes maintained limited and individualized training routines adapted to the imposed restrictions. However, these activities lacked the structured intensity, volume, frequency, and duration typically required for systematic athletic development.

**Suggestion:** Page 15, line 78 – again… How do you know they were unactive? Did you track their behavior somehow?

**Response:** *Due to the COVID-19 pandemic, athletes had to go through a long period of lockdown. As a result, they were unable to follow their regular training routines. We have revised the manuscript to clarify the athletes’ physical activity status during the 14-week COVID-19 lockdown period. We have added an explanation stating that information regarding the athletes’ activity levels during this period was obtained through self-reports collected by the Turkish National Wrestling Team coaches, with one of the coaches also being an author of this study. This addition enhances the reliability of the reported activity levels. We also clarified that during the early phase of the lockdown, the wrestlers were predominantly inactive at home due to the uncertainty surrounding the restrictions, and later gradually engaged in limited individual exercises. These revisions have been incorporated to provide a clearer context regarding the athletes’ activity levels throughout the 14-week period before resuming systematic training.*

“The purpose of this study was to examine the changes in the physical and physiological conditions of elite wrestlers on the Turkish National Wrestling Team who experienced a prolonged period of limited and unstructured physical activity during a 14-week COVID-19 lockdown, followed by an 8-week retraining and competition phase.”

“During the 14-week COVID-19 lockdown period, information regarding the wrestlers’ physical activity levels was obtained through self-reports collected by the Turkish National Wrestling Team coaches. Notably, one of the coaches involved in collecting these reports was also one of the authors of this study, ensuring close oversight and enhancing the reliability of the data. The athletes reported that they stayed at home as a necessity of the pandemic restrictions and engaged only in simple home exercises, walking, and limited jogging activities during this time. They did not follow any structured external (personal) trainer-supported training program, and no specific dietary interventions were applied. In the early phase of the lockdown, the wrestlers were predominantly inactive at home due to the uncertainty surrounding the duration of the restrictions. As the lockdown continued, they gradually began participating in limited individual exercises. Nevertheless, these activities lacked the structured intensity, volume, frequency, and duration typically required for systematic athletic conditioning.”

**Suggestion:** Page 15, line 95 – indicator of what specifically within the ANS? Provide details.

**Response:** *In the revised manuscript, we have clarified that heart rate variability (HRV) specifically reflects the balance between sympathetic and parasympathetic activities within the autonomic nervous system (ANS). Higher HRV is indicative of enhanced parasympathetic modulation and cardiovascular adaptability, whereas lower HRV suggests sympathetic dominance and reduced autonomic flexibility. This additional information has been incorporated to improve the clarity and specificity of the manuscript.*

Heart rate variability (HRV) is a well-established non-invasive indicator of autonomic nervous system (ANS) regulation, specifically reflecting the balance between sympathetic and parasympathetic nervous system activities. Higher HRV is generally associated with increased parasympathetic (vagal) modulation and better cardiovascular adaptability, whereas reduced HRV indicates sympathetic dominance and impaired autonomic flexibility. (Shaffer & Ginsberg, 2017)

**Suggestion:** Page 16, line 106 – which tools have you used to measure these?

**Response:** *Thank you for your comment. This section corresponds to the 'Experimental Approach to the Problem' part of our study. All measurement tools, procedures, and parameters evaluated in the study are described in detail under the relevant subheadings within the Methods section of the manuscript.*

**Suggestion:** Line 18, line 144 – BMI is often times not a relevant measure for athletes, especially for wrestlers many of whom are short and have large muscle mass. Reconsider involving this measurement.

**Response:** *We partially agree with your point that BMI may have limitations when applied to athletic populations, particularly in sports such as wrestling where athletes often have high muscle mass and relatively shorter stature. In our study, BMI was not used as a primary indicator of body composition or health status but rather included to present basic demographic characteristics of the participants. To overcome the known limitations of BMI and to better characterize body composition, we also measured subcutaneous fat thickness (SFT) using standardized anthropometric techniques. Thus, while BMI data is reported for descriptive purposes, body composition assessments were based on more precise and sport-appropriate measurements.*

BMI values were reported for descriptive purposes only and were complemented with direct body composition measurements such as subcutaneous fat thickness (SFT) to better reflect the athletes’ physical profiles.

**Suggestion:** Page 19, line 175 – what is SDNN and RMSSD? Explain before using abbreviations.

**Response:** *We would like to note that the definitions of SDNN (standard deviation of NN intervals) and RMSSD (root mean square of successive RR interval differences) were provided in the 'Experimental Approach to the Problem' section, where these terms were first introduced. These measures are commonly used time-domain parameters in heart rate variability analysis, and we ensured that they were clearly explained upon their initial mention.*

**Suggestion:** Page 20, line 195 – this is repetitive… you’ve already said when the measurements were taken

**Response:** *We have revised the sentence in line with your suggestion to avoid repetition.*

**Suggestion:** Page 21, 208 -again repetitive, you’ve already stated this about your sample

**Response:** *We have revised the sentence in line with your suggestion to avoid repetition.*

**Suggestion:** Page 21, line 209-10 – which measurements specifically?

**Response:** *In response, we have incorporated additional results related to the biweekly changes in body composition parameters, including body fat percentage, muscle mass, and BMI. Table 1 summarizes the progression of these variables over the 8-week retraining period. Statistically significant improvements were observed in body fat percentage and muscle mass at each successive measurement point compared to the previous assessment. Furthermore, we have added detailed results regarding the biweekly changes in subcutaneous fat thickness measurements in the Results section, as described below. These additions provide a clearer and more comprehensive understanding of the improvements in body composition parameters during the retraining phase.*

“Anthropometric assessments were conducted biweekly during the training period. Subcutaneous fat thickness was measured at the triceps, abdominal, subscapular, supraspinale, pectoral, midaxillary, and quadriceps regions using the skinfold method. In addition, body fat percentage and muscle mass were evaluated using the bioelectrical impedance analysis (BIA) method. Statistically significant improvements were observed in body composition parameters with each successive assessment compared to the previous measurement point. (Table 1).

Table 1. Biweekly Changes in Body Weight, Body Fat Percentage, Muscle Mass, and BMI During the Retraining Period”

“There was a time x group interaction in subcutaneous fat thicknesses. Significant reductions were observed in subcutaneous fat thickness measurements throughout the 8-week retraining period (Figure 2). Triceps skinfold thickness decreased significantly at each measurement point compared to the previous one (p < 0.01). Similarly, abdominal (p < 0.001), supraspinale (p < 0.001), midaxillary (p < 0.01), subscapular (p < 0.001), and quadriceps (p < 0.01) regions demonstrated significant decreases at every assessment. In the pectoral region, a significant reduction was observed at week 2 (p < 0.01) and week 4 (p < 0.05); however, no further significant changes were noted thereafter. These findings collectively indicate a consistent improvement in body composition during the retraining phase, as reflected by reductions in subcutaneous fat thickness across most measured sites.”

**Suggestion:** Your study is not cross-sectional, but rather longitudinal because you have several measurements over a period of time.

**Response:** *In line with your suggestion, we have corrected the description of the study design as longitudinal. We have revised the sentence as follows:*

This study is a longitudinal investigation aiming to analyze the potential alterations in the physical, physiological, and performance metrics of elite senior national wrestlers competing in the Greco-Roman style.

**Suggestion:** Another major limitation to your study is convenience sampling.

**Response:** *Due to the constraints of the COVID-19 period, access to participants was significantly limited, which made convenience sampling the most feasible approach under the circumstances. In response to your suggestion, we have acknowledged this as a limitation in the manuscript. Specifically, we have added the following statement to the Limitations section:*

“Additionally, the study utilized convenience sampling, as access to participants was severely restricted during the COVID-19 pandemic. Although convenience sampling was the most feasible approach under these circumstances, it may limit the generalizability of the findings. Future studies should consider employing randomized or stratified sampling methods to enhance sample representativeness and strengthen the robustness of the conclusions.”

**Suggestion:** You are missing a table that describes your participants.

**Response:** *We provided a detailed description of the participants within the text, specifying their number, age, athletic background, competitive level, and major achievements. This information is presented in the Methods section under the Participants subsection. Given the relatively small sample size and the detailed narrative description already included, a separate table summarizing participant characteristics was not initially created.*

**Suggestion:** A native English-speaking person reading and adjusting this text would be of great value.

**Response:** *In response, we have carefully revised the entire manuscript to enhance the clarity, fluency, and academic quality of the English language. Additionally, the manuscript was reviewed and edited with the assistance of an advanced native-level English language model (ChatGPT-4o, OpenAI), followed by final adaptations made by the authors to ensure accuracy and adherence to scientific writing standards. We believe that these improvements have significantly enhanced the readability and quality of the manuscript.*
