# Supplementary material for: The impact of 8-week re-training following a 14-week period of training cessation on Greco-Roman Wrestlers
Source: PLoS One. 2025 Jun 25;20(6):e0326731. doi: 10.1371/journal.pone.0326731 (PMC12194023; doi:10.1371/journal.pone.0326731)
Supplement: Attachment — Submitted filename: Responso to revievers- 3.docx [file pone.0326731.s003.docx]

**Reviewer #2:**

We sincerely thank you for your valuable comments and suggestions. We have carefully addressed each of your points and revised the manuscript accordingly. Below, we provide a detailed, point-by-point response. The authors’ responses are written in italics. Where appropriate, changes made in the manuscript have been highlighted in yellow.

**Suggestion:** Page 18, line 26 – authors failed to report how long the detraining phase was in the abstract section. If you state that retraining period was 8 weeks, it would be of great value to also know how long was the detraining phase.

**Response:** *Thank you for your valuable comment. As you rightly pointed out, clearly stating the duration of the detraining phase adds clarity and value to the abstract and overall interpretation. Although the 14-week detraining period was already mentioned in the background section in abstract, we have now revised the conclusion section to explicitly include the detraining duration in the relevant sentence for improved clarity. The revised sentence now reads:*

*“Moreover, HRV monitoring revealed that autonomic nervous system balance was compromised during both the 14-week detraining and the subsequent competition phases, underlining the need for careful training load management to optimize recovery and performance readiness.”*

*We hope this revision addresses your concern.*

**Suggestion:** Page 19, line 44 – “During these periods of partial or complete training interruption, it has been suggested that the organism’s responsiveness to exercise is significantly prolonged”. I am not sure what this sentence means. Refine for clarity.

**Response:** *Thank you for your comment. We agree that the original sentence lacked clarity and could be misinterpreted. To improve the precision of the statement, we have revised the sentence on Page 19, line 44 as follows:*

*“During periods of partial or complete training interruption, the body’s physiological responsiveness to exercise stimuli may become diminished or delayed, leading to a slower return to previous performance levels upon retraining.”*

*We believe this updated version more accurately reflects the intended meaning and appreciate your helpful suggestion.*

**Suggestion:** Page 19, line 46 – “Athletes facing training interruptions may encounter risk factors that negatively impact performance, including declines in muscle strength, muscle size, cardiovascular endurance, as well as impairments in neuromuscular coordination and flexibility.” Provide citation(s).

**Response:** *We have added relevant citations to the sentence on Page 19, line 46, which now reads:*

*“Athletes facing training interruptions may encounter risk factors that negatively impact performance, including declines in muscle strength, muscle size, cardiovascular endurance, as well as impairments in neuromuscular coordination and flexibility (Alvurdu et al., 2022; Bosquet et al., 2013; Mujika & Padilla, 2000b).*

**Suggestion:** Page 21, line 79 – “Proper training methods can minimize the risk of injury, prevent long-term losses in muscle size and strength, and facilitate a faster and more reliable return to optimal fitness and performance levels. Structured and individualized retraining programs are crucial for minimizing injury risk and accelerating performance recovery in elite 75 wrestlers following prolonged periods of training cessation. Given the detrimental effects of training cessation on athletic performance, it is essential for athletes to implement appropriate strategies to mitigate performance declines.” Move this section to discussion. It deviates from the key points in the Introduction and it makes too long.

**Response:** *As recommended, we have moved the relevant section from the Introduction to the Discussion to maintain a clearer focus on the key points and improve the overall structure and flow of the manuscript. We believe this adjustment enhances both the coherence and readability of the paper.*

**Suggestion:** Page 24, line 148 – which device did you use to measure HRV?

**Response:** *We have revised the sentence to include the specific device used for HRV measurement. The updated sentence now reads:*

*“HRV data were collected using a Polar H10 heart rate chest strap sensor.”*

**Suggestion:** Page 24, line 157 – Turkish national team I’d assume? Mention this. It’s important.

**Response:** *We have revised the sentence to clearly indicate that the participants were members of the Turkish National Wrestling Team. The updated sentence now reads:*

“*This study included 20 male volunteer wrestlers (aged 21.75 ± 3.11 years) who were members of the Turkish National Wrestling Team and had been training for at least 10 years.”*

**Suggestion:** Page 26, line 199 – they COULD not… Stay in the past tense.

**Response:** *As suggested, we have revised the sentence to remain consistent with the past tense. The corrected version now reads:*

*“Due to the constraints imposed by the pandemic, the wrestlers unable to perform a structured training program during the long-term training cessation period.”*

**Suggestion:** Page 26, line 213 – WERE required. The same principle applies.

**Response:** *As suggested, we have revised the sentence to remain consistent with the past tense. The corrected version now reads:*

*“Wrestlers were required to attend at least 90 % of the training sessions. The weekly breakdown of the 8-week training program is provided in Table 1.”*

**Suggestion:** Page 28, line 239 – “The participant was performed three sets”.

**Response:** *As suggested, we have revised the sentence to remain consistent with the past tense. The corrected version now reads:*

*“Each participant performed three sets of 30-second dummy throws, separated by 20-second rest intervals.”*

**Suggestion:** As I said in the initial review, this manuscript would benefit greatly if the native English-speaking person reads it.

**Response:** *As per your suggestion, the manuscript has been thoroughly reviewed and revised by a native English speaker to improve its linguistic quality, clarity, and fluency.”*
